# HIV prevalence among men who have sex with men, transgender women and cisgender male sex workers in sub‐Saharan Africa: a systematic review and meta‐analysis

**DOI:** 10.1002/jia2.26022

**Published:** 2022-11-23

**Authors:** Mariëlle Kloek, Caroline A. Bulstra, Laura van Noord, Lina Al‐Hassany, Frances M. Cowan, Jan A. C. Hontelez

**Affiliations:** ^1^ Department of Public Health Erasmus MC, University Medical Center Rotterdam Rotterdam The Netherlands; ^2^ Heidelberg Institute of Global Health Medical Faculty and University Hospital Heidelberg University Heidelberg Germany; ^3^ Centre for Sexual Health and HIV AIDS Research (CeSHHAR) Zimbabwe Harare Zimbabwe; ^4^ Department of International Public Health Liverpool School of Tropical Medicine Liverpool UK

**Keywords:** HIV, prevalence, sub‐Saharan Africa, men who have sex with men, transgender, male sex workers

## Abstract

**Introduction:**

Developing effective targets, policies and services for key populations requires estimations of population sizes and HIV prevalence across countries and regions. We estimated the relative and absolute HIV prevalence among men who have sex with men (MSM), transgender women and men, and male and transgender sex workers (MSW and TGSW) in sub‐Saharan African countries using peer‐reviewed literature.

**Methods:**

We performed a systematic review of peer‐reviewed studies assessing HIV prevalence in MSM, transgender women and men, MSW and TGSW in sub‐Saharan Africa between 2010 and 2021, following PRISMA guidelines. We searched Embase, Medline Epub, Africa Index Medicus, Africa Journal Online, Web of Science and Google Scholar. We calculated HIV prevalence ratios (PRs) between the study prevalence, and the geospatial‐, sex, time and age‐matched general population prevalence. We extrapolated results for MSM and transgender women to estimate HIV prevalence and the number living with HIV for each country in sub‐Saharan Africa using pooled review results, and regression approximations for countries with no peer‐reviewed data.

**Results and discussion:**

We found 44 articles assessing HIV prevalence in MSM, 10 in transgender women, five in MSW and zero in transgender men and TGSW. Prevalence among MSM and transgender women was significantly higher compared to the general population: PRs of 11.3 [CI: 9.9–12.9] for MSM and 8.1 [CI: 6.9–9.6] for transgender women in Western and Central Africa, and, respectively, 1.9 [CI: 1.7–2.0] and 2.1 [CI: 1.9–2.4] in Eastern and Southern Africa. Prevalence among MSW was significantly higher in both Nigeria (PR: 12.4 [CI: 7.3–21.0]) and Kenya (PR: 8.6 [CI: 4.6–15.6]). Extrapolating our findings for MSM and transgender women resulted in an estimated HIV prevalence of 15% or higher for about 60% of all sub‐Saharan African countries for MSM, and for all but two countries for transgender women.

**Conclusions:**

HIV prevalence among MSM and transgender women throughout sub‐Saharan Africa is alarmingly high. This high prevalence, coupled with the specific risks and vulnerabilities faced by these populations, highlights the urgent need for risk‐group‐tailored prevention and treatment interventions across the sub‐continent. There is a clear gap in knowledge on HIV prevalence among transgender men, MSW and TGSW in sub‐Saharan Africa.

## 1 INTRODUCTION

Sub‐Saharan Africa (SSA) is the epicentre of the HIV pandemic, with about 21 million people living with HIV [1]. Especially countries in Eastern and Southern Africa (ESA) are faced with so‐called generalized epidemics, affecting large parts of the general population, while HIV prevalence in Western and Central African (WCA) countries is mostly concentrated among people at higher risk for HIV [1]. The successful rollout of HIV treatment and prevention programmes across the subcontinent over the past decades has curbed transmission among the general population and female sex workers in many settings [2, 3, 4]. However, stigma and criminalization cause barriers to access for other key populations, such as cisgender men who have sex with men (MSM), transgender people, and cisgender male and transgender sex workers (MSW and TGSW) [5, 6, 7, 8, 9]. Currently, an estimated 54% of all new HIV infections worldwide occur among key populations and their sex partners [10], and compared to the general population, the average risk for HIV infection is about 20 times higher for sex workers and MSM, and about 10 times higher for transgender people [10]. For MSM, particular risk factors include condomless anal sex, discrimination and criminalization in many SSA settings [11, 12], while for transgender people, further HIV risks are added due to needle sharing for hormonal therapy, and transgender people are particularly vulnerable for social isolation and stigma in many countries [12, 13]. Male and TGSWs are additionally faced with the increased risks of being engaged in commercial sex, that is having many sexual partners [8, 13].

Developing effective targets, policies and interventions requires estimations of population sizes and HIV prevalence across countries and regions [14]. Furthermore, such information could improve our understanding of the relative importance of these key populations in the overall epidemic, thereby improving mathematical modelling projections on the impact of interventions for each key population and for the general epidemic. However, current population size and HIV prevalence estimates for MSM provided by the Joint United Nations Programme on HIV/AIDS (UNAIDS) largely rely on country‐reported numbers from a single survey or expert opinion—and are potentially biased [15]—while estimates for transgender people, and MSW and TGSW are mostly completely absent. Summarizing and extrapolating HIV prevalence estimates from the recent existing scientific literature to estimate country‐specific HIV prevalences for these key populations in sub‐Saharan Africa could help fill this gap in knowledge, by improving our understanding of the current HIV prevalence among these populations. While previous systematic reviews have included a limited number of studies from sub‐Saharan Africa [11, 16, 17, 18], these reviews have been conducted almost a decade ago. Several studies have been published since then, and current prevalence is likely very different from the time those reviews were conducted, as the rapid scale‐up of antiretroviral therapy (ART) and other prevention interventions across the subcontinent has substantially changed HIV epidemiology over the past decade.

The aim of this study was to estimate the recent relative and absolute HIV prevalence for MSM, transgender women and men, MSW and TGSW in sub‐Saharan Africa. We first systematically reviewed peer‐reviewed studies on the prevalence of HIV in each of these key populations, and then estimated the relative HIV prevalence by comparing prevalence estimates to geospatial‐, sex, time and age‐matched estimates of HIV prevalence in the general population. We then applied pooled estimates of relative risk and prevalence to country‐specific HIV epidemics to estimate the country‐specific HIV prevalence per risk group for each country in sub‐Saharan Africa.

## 2 METHODS

### 2.1 Search strategy and selection criteria

We followed PRISMA guidelines for systematic reviews and meta‐analyses [19]. We searched Embase, Medline Epub, Africa Index Medicus, Africa Journal Online, Web of Science and Google Scholar to identify studies that report HIV prevalence among MSM, transgender women and men, MSW and/or TGSW in sub‐Saharan African countries, in peer‐reviewed literature, reporting on data collected between 1 January 2010 and 22 October 2021. We choose this time period to strike a balance between the accuracy of our estimates of the current prevalence and relative risks in each key population versus the power to perform any meaningful meta‐analyses. We constructed search strings in collaboration with a medical librarian (see File S1 in the Appendix for the complete search strategy). We used Medical Subject Headings (MeSH) terms and “all fields” terms comprising sex work (“sex worker,” “prostitute”), LGBT people (“MSM,” “transgender,” “gay”), HIV/AIDS, prevalence (“cross‐sectional study,” “incidence,” “odds ratio”) and sub‐Saharan Africa. After an initial search in June 2018, the search has been updated in July 2020 to include the most recent publications, and again in October 2021 to include more recent publications and the regional databases Africa Index Medicus and Africa Journal Online. In both cases, no changes were made to the search terms.

We included peer‐reviewed studies that reported HIV prevalence or data from which HIV prevalence could be derived among MSM, transgender people, MSW and/or TGSW, of a site in at least one country in sub‐Saharan Africa, had a cross‐sectional or cohort study design and were published in English or French. We excluded studies that: [1] were based on self‐reported HIV status; [2] assessed subgroups of the study population (i.e. prisoners, drug‐using MSM, MSW among an MSM population), as estimates from such subgroups are likely biased towards higher HIV prevalence levels, making them not generalizable to the entire study population; [3] were a secondary analysis of previously collected data; [4] did not provide a prevalence estimate; [5] were based on data collected before 2010; and [6] failed to correctly define the different key populations (see panel S1 in Appendix). Different studies conducted at the same location were included to maximize power, except if they were based on the same dataset. In that case, we included the study presenting the greatest total number of people tested.

Two independent reviewers (MK and LvN) performed a screening of titles and abstracts of retrieved records. For those deemed eligible based on the set inclusion and exclusion criteria, full texts were examined to determine full eligibility. Any disagreements between the independent reviewers were resolved by consensus with the senior author (JACH).

### 2.2 Data extraction and meta‐analyses

Two authors independently extracted the following study characteristics: population studied, study location, study year, study design, recruitment method, number of participants, age distribution, type of HIV test and HIV prevalence with 95% confidence intervals (CIs). When the HIV prevalence and/or 95% CIs were not reported directly, we calculated these using the reported absolute numbers. The corresponding authors of studies were contacted if additional study information was required. The risk of bias was assessed using Joanna Briggs Institute (JBI) critical appraisal checklist (Table S2 in Appendix) [20].

For each study, we calculated a prevalence ratio (PR) of HIV prevalence in the key population of interest, compared to the HIV prevalence in the general population. We derived general population HIV prevalence data from Demographic Health Surveys (DHSs) and AIDS Indicator Surveys (AISs), which are nationally representative household surveys that often include voluntary HIV testing in adults, and have been systematically performed in many countries in sub‐Saharan Africa [21]. Typically, a DHS or AIS is performed at around 350 randomly selected sample locations in each country, and all members of about 25 households at each location are invited to participate [22]. These data were the only available general population HIV prevalence data that could be geospatially matched with locations of the studies in our review, and are generally assumed to be fairly representative of general population‐level HIV prevalence estimates [23, 24].

For each study in our review, we first geo‐located the study site, and then selected DHSs/AISs sample locations from the survey conducted closest to the year of data collection in the study, with a maximum difference of 3 years. If, after contacting corresponding authors, the year of data collection was still missing, the most reasonable DHS/AIS was selected based on the publication date of the study. We selected all sample locations within a 5‐kilometre radius from the study site, and calculated the general population HIV prevalence in the selected sample locations, standardized to the study population by age composition and gender (i.e. only males when comparing to MSM and MSW prevalence, and males and females when comparing to transgender women).

For studies without DHS/AISs data collected within 3 years before or after the study, we extracted local HIV prevalence estimates from the study by Dwyer‐Lindgren et al. [25]. They estimated yearly 5 by 5‐kilometre HIV prevalence for the whole of sub‐Saharan Africa from 2000 to 2017, for females and males (15–49 years) combined. They estimated HIV prevalence based on a variety of data sources, including local studies, antenatal care surveys and population‐based surveys, and age‐ and sex‐standardization was not possible with these data.

We calculated the PR as the ratio between the prevalence among the key population in the study and the prevalence in the general population at that location. A pooled prevalence and PR, stratified by country and region (WCA and ESA), was calculated by summing absolute numbers of all studies and calculating a combined prevalence and PR. We stratified by region to control for potential effect modification, as PRs may differ for the more concentrated epidemics in WCA versus the mixed and generalized epidemics in ESA.

For MSM and transgender women, the total number of studies identified allowed us to extrapolate HIV prevalence derived from our review to crudely estimate the country‐specific prevalence in 5 percentage point intervals (0–5%, 5–10%, 10–15%, 15–20% and >20%) for all countries in sub‐Saharan Africa. For the countries for which we had data and a sufficient number of people tested (*n*≥80 for MSM and *n*≥50 for transgender people), we divided them into the prevalence categories using the pooled country estimated prevalence. For the countries for which we had no data or an insufficient number of people tested (*n*<80 for MSM and *n*<50 for transgender people), we estimated the HIV prevalence in MSM and transgender women through a regression approximation. The cut‐off values of 80 and 50 participants, respectively, were arbitrarily chosen to ensure that studies with very small sample sizes would not dilute our regression analyses, and to ensure that countries would not be categorized based on a single study with a very small sample size.

The regression approximation was performed as follows. We first determined the relationship between the HIV prevalence in the study population and general HIV prevalence for all studies in our review by fitting a logistic Deming regression, with the HIV prevalence in the studies as the dependent variable, and corresponding HIV prevalence in the general population as the independent variable. We then applied this function to the country‐level general urban population HIV prevalence for countries for which we had insufficient peer‐reviewed data, as all peer‐reviewed studies were conducted in urban settings. The general urban population HIV prevalence for each country was estimated by multiplying general population HIV prevalence estimates derived from UNAIDS 2020 [26] with a country‐specific ratio of urban total HIV prevalence derived from DHS [21], and an average urban total HIV prevalence over all countries for countries without DHS data. The function for MSM is log odds(*y*) = –1.96 + 0.021*x* (*p* = 0.07), and the function for transgender women is log odds(*y*) = –1.64 + 0.059*x* (*p* = 0.05), where *y* = HIV prevalence in the key population, and *x* = HIV prevalence in the general population. We did not stratify our regression approximation by region. Yet, the regression models inherently capture prevalence heterogeneities across the regions, as the model is fitted using general population HIV prevalence as a predictor.

After estimating the relative HIV prevalence for each country, we roughly estimated the country‐specific absolute HIV prevalence. We first applied estimates of the proportion of MSM (1.0–4.0%) and transgender women (0.5–1.0%) within populations [27] to the United Nations population size estimates [28] to develop rough population size estimates for the key populations. We then multiplied these with prevalence estimates for the key population, assuming rural and urban HIV prevalence levels to be the same. See Appendix panel S2 for a detailed description of the applied approach.

We performed several sensitivity and validation analyses on our prevalence estimations. First, we determined the impact of preferring DHS data over data from Dwyer‐Lindgren et al. [25] as a source for HIV prevalence in the local general population by running our analyses using both DHS and Dwyer‐Lindgren et al. [25] data for studies where this was possible and compared the resulting PRs. Second, we tested the validity of our regression approximation by applying the model to countries where we had used pooled peer‐reviewed data to estimate the country‐level prevalence and compared the outcomes. Third, we tested whether the year of data collection, legal or illegal status of same‐sex relationships and an indicator for the severity of anti‐LGBT laws [30] could explain some of the observed heterogeneity in the relationship between key and general population HIV prevalence by testing them as predictors in a logistic regression model. Fourth, we determined whether UNAIDS [26] reported point estimates of prevalence, based on grey literature, fell within the prevalence category assigned to each country based on our estimates.

All analyses were done using R version 4.0.0 and ArcGIS Pro version 2.5.0.

### 2.3 Role of funding source, interests and registration

The study's funder had no role in study design, data collection, data analysis, data interpretation, writing or submitting of the report. Independent authors declare no competing interests. The review was not registered.

## 3 RESULTS AND DISCUSSION

**Figure 1:**
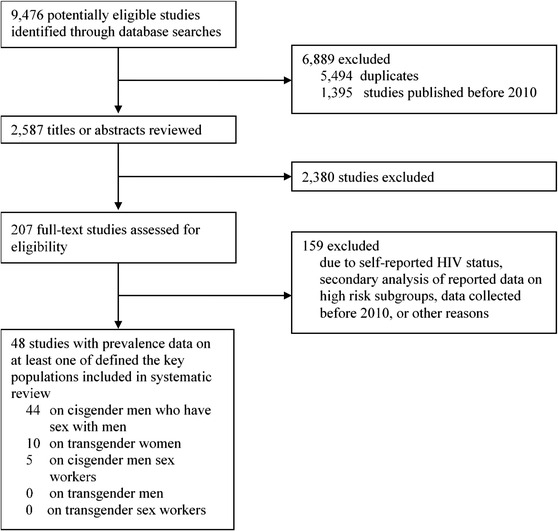
Flow chart of study selection disposition.

**Figure 2:**
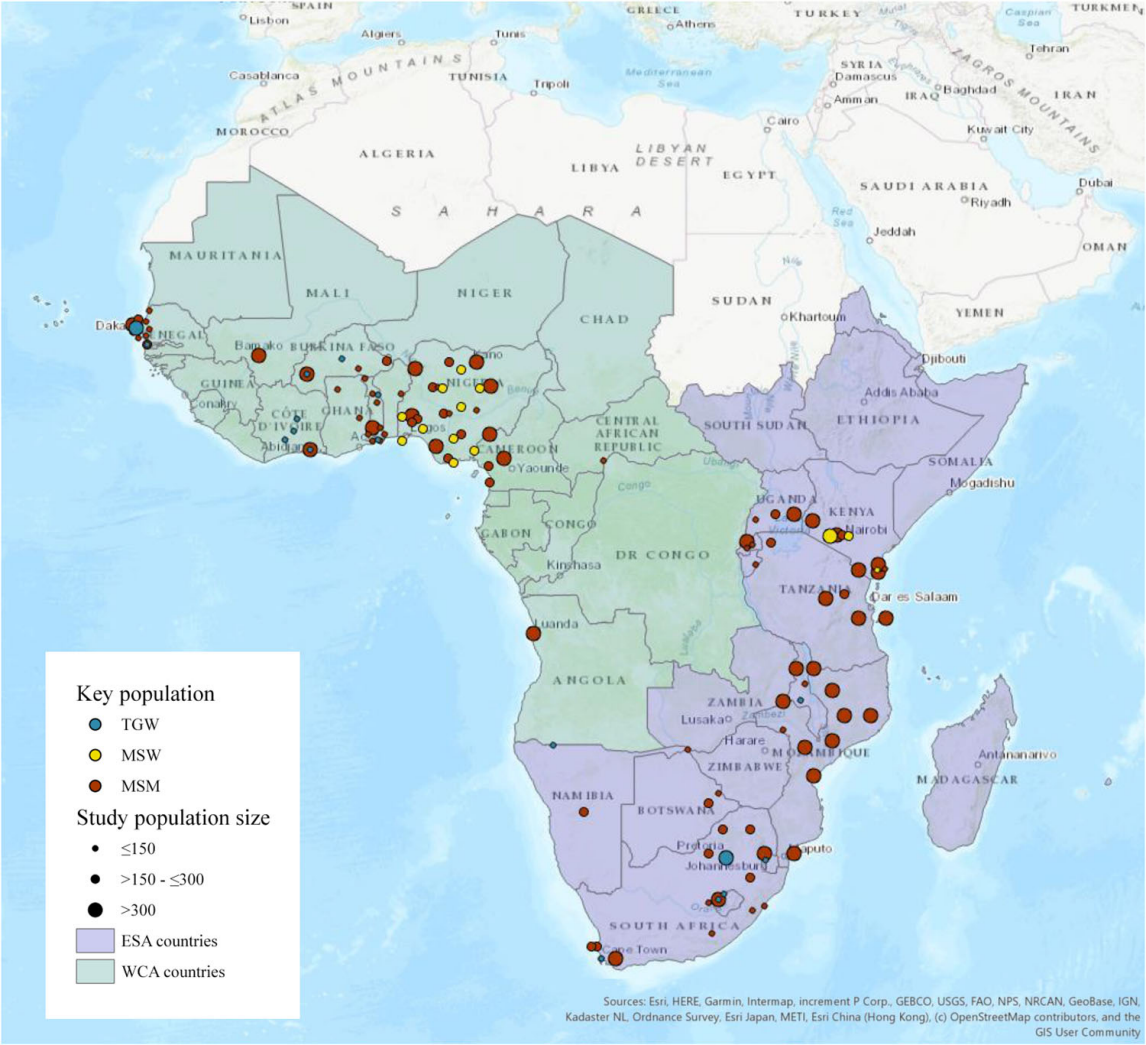
Locations of the included studies on HIV prevalence in men who have sex with men (MSM), transgender women (TGW) and male sex workers (MSW) in sub‐Saharan Africa. Abbreviations: ESA countries, Eastern and Southern African countries; WCA countries, Western and Central African countries.

Our search identified 9476 articles, of which 2587 were unique records (Figure 1). Based on the screening of the title and abstract, 207 full texts were retrieved, of which 48 met our inclusion criteria [12, 31, 32, 33, 34, 35, 36, 37, 38, 39, 40, 41, 42, 43, 44, 45, 46, 47, 48, 49, 50, 51, 52, 53, 54, 55, 56, 57, 58, 59, 60, 61, 62, 63, 64, 65, 66, 67, 68, 69, 70, 71, 72, 73, 74, 82, 83, 84, 85]. Most studies were excluded because they either only collected self‐reported HIV status, re‐analysed previously collected data that was already part of the review or provided data collected pre‐2010. We did not find studies with conflated gender group definitions. A complete overview of each literature search is given in Table S1. The majority of publications (91.7%, 44/48) provided HIV prevalence data on MSM (Table S3 in Appendix), compared to 20.8% (10/48) on transgender women (Table S4 in Appendix) and 10.4% (5/48) on MSW (Table S5 in Appendix). No articles were identified with relevant data on transgender men or TGSW. The 48 articles covered 21 of the 47 sub‐Saharan African countries (44.7%); 10 in WCA and 11 in ESA (Figure 2). The five studies that assessed HIV prevalence in MSW covered only two countries: Nigeria and Kenya [40, 49, 72, 73, 74]. All studies were performed in urban settings, and all were deemed of sufficient quality based on the JBI critical appraisal checklist (see Appendix Table S2).

**Figure 3:**
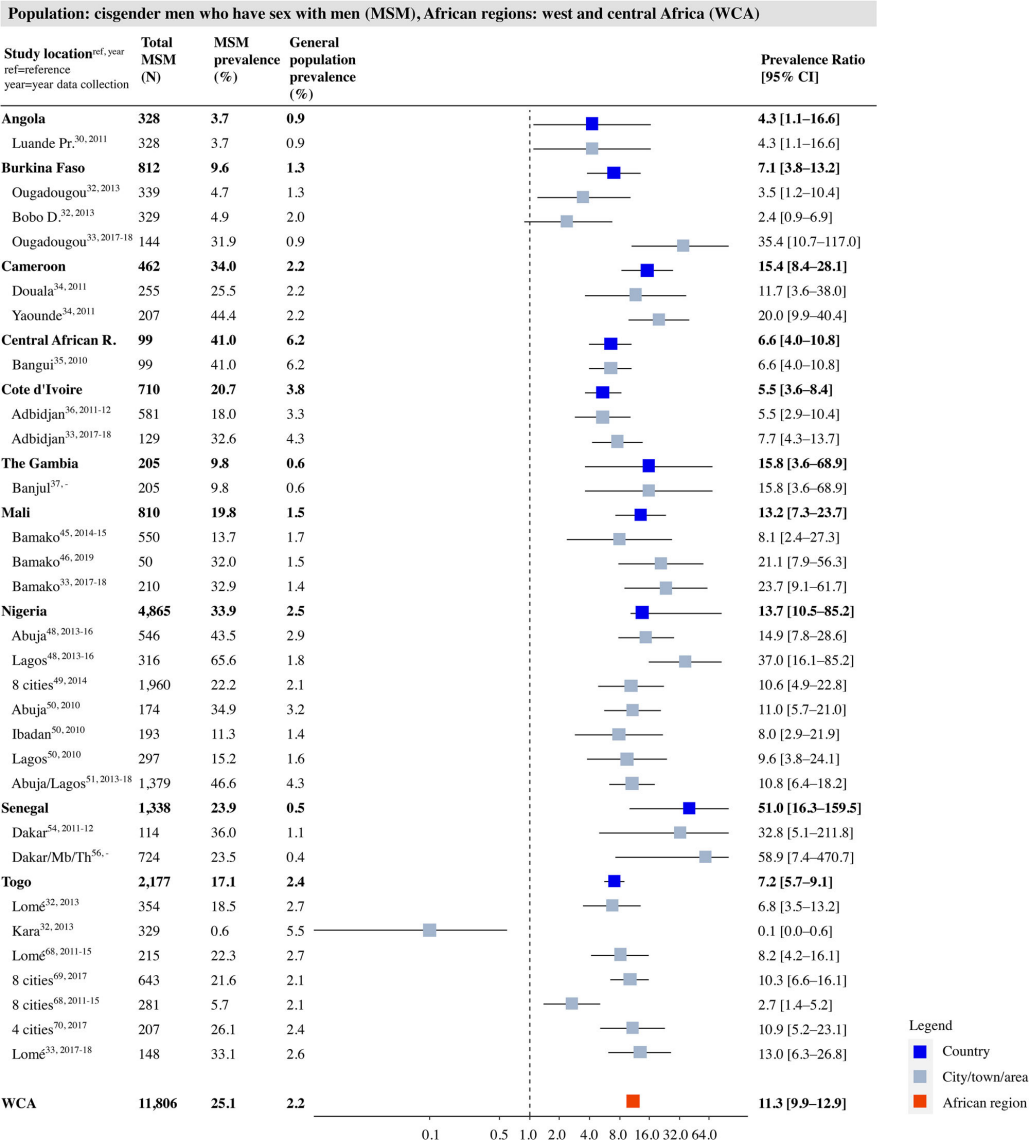
HIV prevalence and prevalence ratio (PR) for men who have sex with men (MSM) per study place, country and region in the West and Central Africa (WCA). Grey squares represent individual study locations, and weighted averages for country and region levels are in blue and red. PRs are relative risks compared to the geospatially matched general male population aged 15–49. Abbreviations: 95% CI, 95% confidence interval; MSM, men who have sex with men.

**Figure 4:**
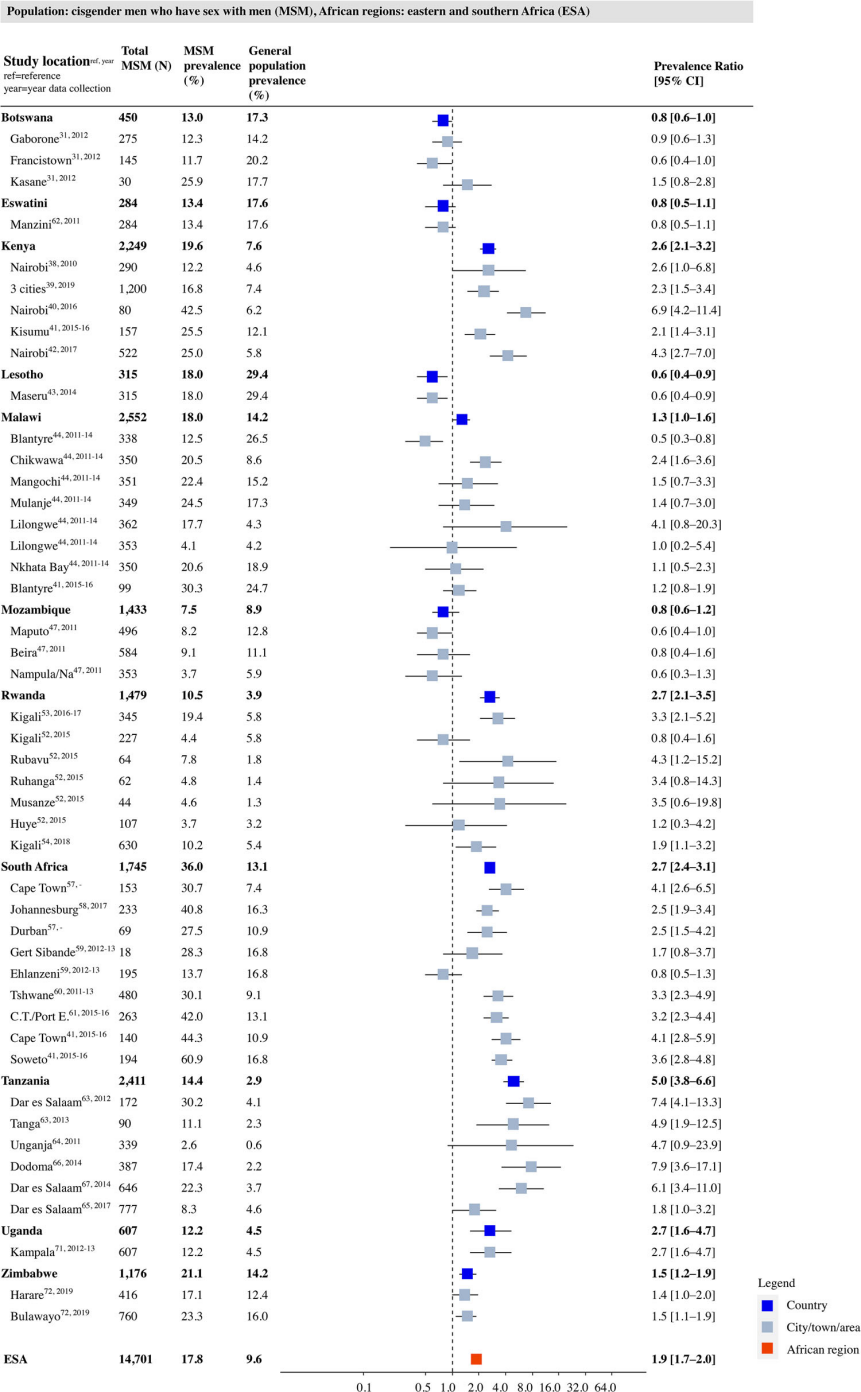
HIV prevalence and prevalence ratio (PR) for men who have sex with men (MSM) per study place, country and region in Eastern and Southern Africa (ESA). Grey squares represent individual study locations, and weighted averages for country and region levels are in blue and red. PRs are relative risks compared to the geospatially matched general male population aged 15–49. Abbreviations: 95% CI, 95% confidence interval; MSM, men who have sex with men.

Study‐, country‐ and region‐specific PRs for HIV in MSM, compared to the general male population, are shown in Figure 3 for WCA and in Figure 4 for ESA. The reported HIV prevalence among MSM in WCA ranged between 4.3% in Angola and 51.0% in Senegal. Prevalence was significantly higher in 27 of the 29 study locations, with a weighted average PR of 11.3 (95% CI: 9.9–12.9). In ESA (Figure 4), the prevalence ranged from 7.5% in Mozambique to 36.0% in South Africa. Only 24 out of the 46 study locations showed a significantly higher HIV prevalence among MSM, with a weighted average PR of 1.9 (95% CI: 1.7–2.0).

**Figure 5:**
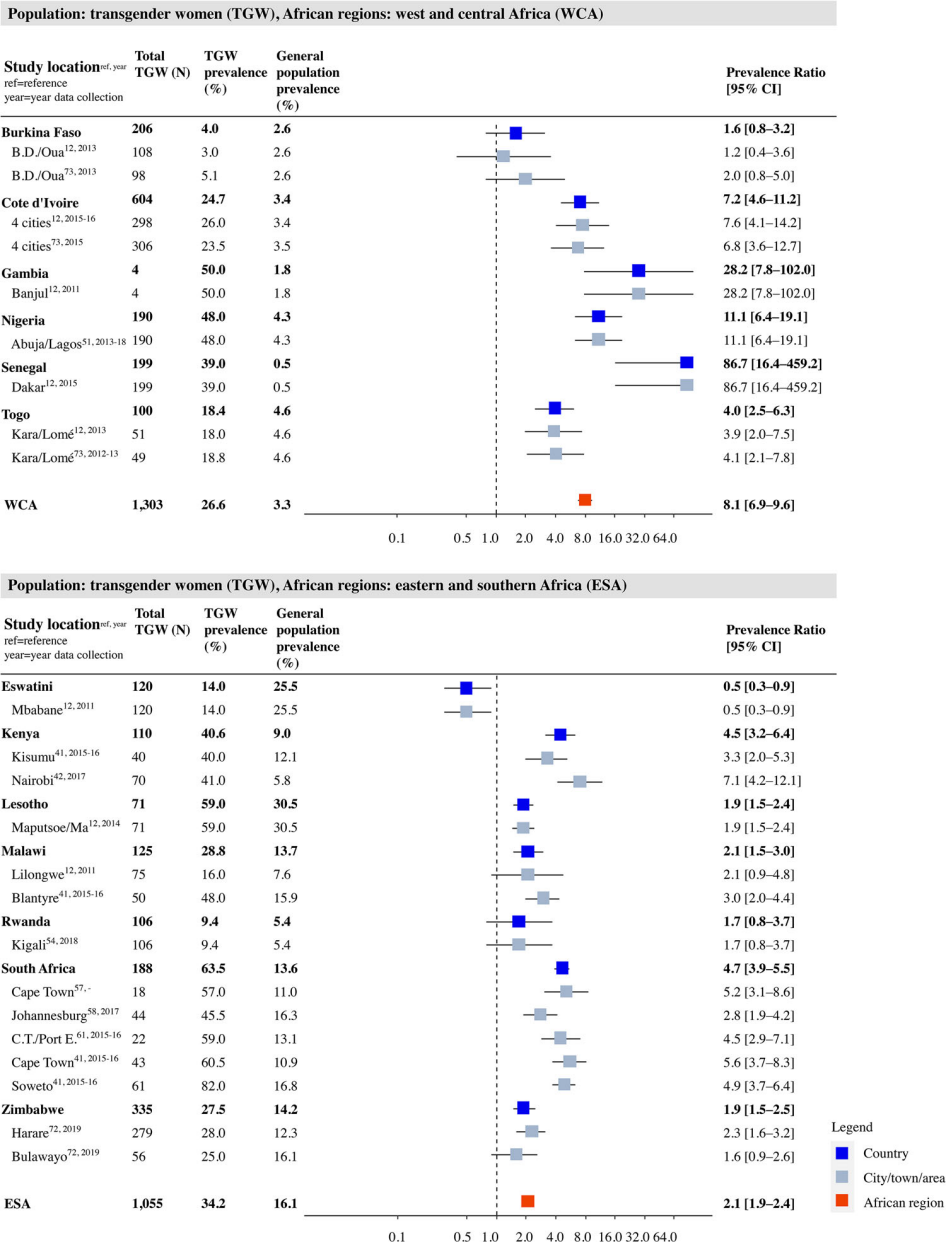
HIV prevalence and prevalence ratio (PR) for transgender women (TGW) per study place, country and region in West and Central Africa (WCA) and Eastern and Southern Africa (ESA). Grey squares represent individual study locations, and weighted averages for country and region levels are in blue and red. PRs are relative risks compared to the geospatially matched general population aged 15–49. Abbreviations: 95% CI, 95% confidence interval; ESA, Eastern and Southern Africa; TGW, transgender women; WCA, Western and Central Africa.

Study‐, country‐ and region‐specific PRs for transgender women are shown in Figure 5. In WCA, the prevalence ranged from 4.0 in Burkina Faso to 50.0 in the Gambia. Seven out of nine study locations showed significantly higher HIV prevalence among transgender women, with PRs ranging from 1.6 to 86.7 (upper panel in Figure 5]. The weighted average PR was 8.1 (95% CI: 6.9–9.6). For ESA, the prevalence ranged from 9.4% in Rwanda to 63.5% in South Africa. Ten out of 14 study locations showed a significantly higher prevalence among transgender women, with PRs ranging from 0.5 to 4.7 and a weighted average PR of 2.1 (95% CI: 1.9–2.4; lower panel in Figure 5].

**Figure 6:**
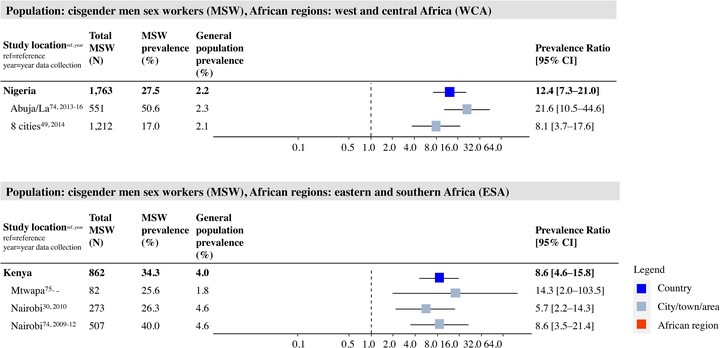
HIV prevalence and prevalence ratio (PR) for male sex workers (MSW) per study place, country and region in West and Central Africa (WCA) and Eastern and Southern Africa (ESA). Grey squares represent individual study locations, and weighted averages for country and region levels are in blue and red. PRs are relative risks compared to the geospatially matched general male population aged 15–49. Abbreviations: 95% CI, 95% confidence interval; ESA, Eastern and Southern Africa; MSW, male sex worker; WCA, Western and Central Africa.

Study‐, country‐ and region‐specific PRs for MSW are shown in Figure 6. For MSW, all five study locations showed a significantly higher prevalence compared to the general population, with an overall PR of 8.6 (95% CI 4.6; 15.8) for Nigeria (upper panel of Figure 6] and 12.4 (95% CI: 7.3–21.0) for Kenya (lower panel of Figure 6].

**Figure 7:**
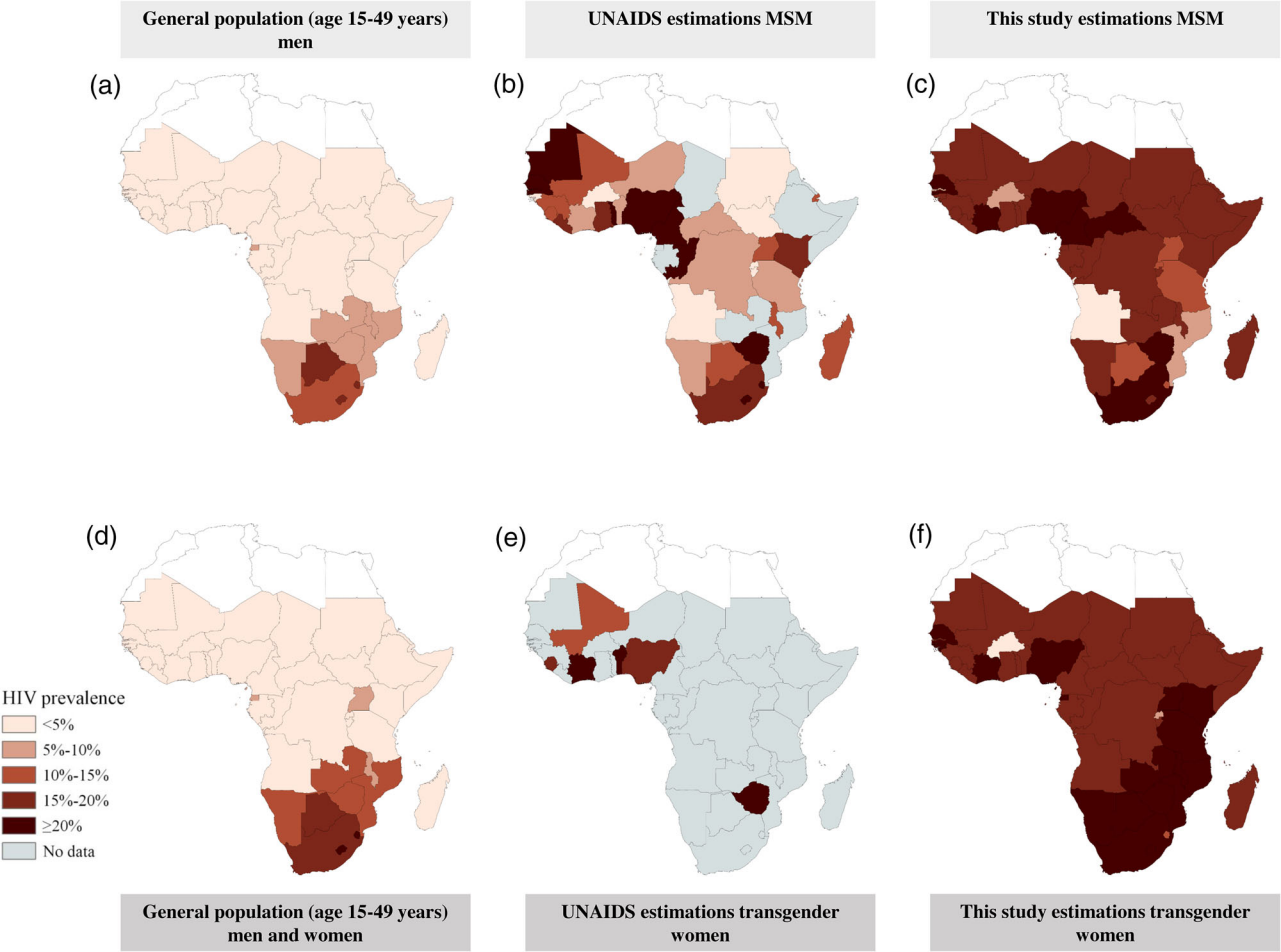
Maps of country‐level HIV prevalence levels for the general population (left column), UNAIDS reported prevalence in MSM and transgender women (middle column) and estimated HIV prevalence among MSM and transgender women based on peer‐reviewed literature (right column). The peer‐reviewed literature estimations are based on the country‐level weighted HIV prevalence derived from the included studies in the systematic literature search. For countries for which we did not find sufficient data, we estimated the country‐level prevalence using a logistic Deming regression model fitted to the relationship between the key population and general population HIV prevalence in peer‐reviewed literature.

We had sufficient data points for both MSM and transgender women to extrapolate our findings to estimate HIV prevalence for the two populations in countries in sub‐Saharan Africa for which we found no studies, using a regression approximation (Figures S1 and S2; Tables S6–S9). The resulting estimated country‐specific HIV prevalences among MSM and transgender women for all countries in sub‐Saharan Africa are presented in Figure 7c and f and are compared to general population prevalence (Figure 7a and d) and UNAIDS estimations (Figure 7b and e). For MSM, we estimated an HIV prevalence of 15–20% for 31 out of the 47 countries (66%) and a prevalence of ≥20% for seven countries (15%). For transgender women, the estimated HIV prevalence was above 15% for all but two countries and was ≥20% for 16 out of 47 countries (34%). For only 11 out of the 37 countries (30%), the UNAIDS point estimate fell within the prevalence categories assigned to those countries in our study (Figure 7 and Appendix Tables S12 and S13). For most countries (20 out of 37), our estimated HIV prevalence for MSM was higher than those published by UNAIDS. Two of the seven countries with UNAIDS reported prevalence data on transgender women. Two matched our estimates, three were higher and two were lower. Our estimations roughly translate into about 600,000–2.2 million MSM and 400,000–800,000 transgender women currently living with HIV in sub‐Saharan Africa (see Appendix Tables S10 and S11 for more details).

Our sensitivity analysis showed that the impact of choosing DHS data over data from Dwyer‐Lindgren et al. had little impact on estimated PRs, as none were significantly different in settings where we could do both (see Appendix Table S16). Furthermore, the year of the survey was borderline significantly associated with a higher HIV prevalence among MSM (*p* = 0.04) and not significant for TGW (*p* = 0.12), while the legal status of same‐sex relationships and severity of anti‐LGBT laws were not significantly associated for both MSM (*p* = 0.9 and 0.5, respectively) and TGW (*p* = 0.08 and 0.3, respectively) (Appendix Tables S7 and S9). When validating regression approximations against data‐based country prevalence estimates, we found that only about 20% of countries would end up in the same prevalence category (see Appendix Tables S14 and S15).

## 4 DISCUSSION

Our systematic review identified 44 articles assessing HIV prevalence in MSM, 10 in transgender women, five in MSW and zero in transgender men and TGSW in sub‐Saharan Africa since 2010. Prevalence among MSM and transgender women was significantly higher than the general population, with PRs for MSM and transgender women ranging from 11.3 and 8.1, respectively, in WCA, to 1.9 and 2.1 in ESA. Furthermore, the prevalence among MSW was also significantly higher in both Nigeria (PR: 12.4 [CI: 7.3–21.0]) and Kenya (PR: 8.6 [CI: 4.6–15.6]), the only two countries with data on MSW. Extrapolating our findings to country‐ and region‐specific estimates resulted in an estimated HIV prevalence of 15–20% among MSM for roughly half of the sub‐Saharan African countries and seven countries with an estimated HIV prevalence of ≥20%. For transgender women, we estimated an HIV prevalence of 15–20% or ≥20% for all but two countries. These estimates roughly translate into about 600,000–2.2 million MSM and 400,000–800,000 transgender women currently living with HIV in sub‐Saharan Africa.

Our study is a major update of earlier reviews of studies on the HIV prevalence among MSM and transgender women in sub‐Saharan Africa [11, 20, 21, 22]. In addition, we are the first to extrapolate geospatial, age, time and sex‐matched associations with the general population HIV prevalence in each study to estimate the HIV prevalence among MSM and transgender women in all countries in sub‐Saharan Africa. It is encouraging that our findings on the PR in sub‐Saharan Africa are consistent with those from Hessou et al. [73] for MSM (a PR for Western Central Africa of 14.5 vs. 11.3 in our study, and 3.4 for Eastern Africa and 1.2 for Southern Africa vs. 1.9 for Eastern Southern Africa in our study), and in line with global estimates on HIV prevalences among transgender people [21].

Our estimates and extrapolations are important when assessing country‐level needs and targets for key population‐specific services, and estimating the required resources to meet those needs and targets. Annual HIV epidemic updates published by UNAIDS [26] provide HIV prevalence estimates for the majority of sub‐Saharan African countries on MSM, usually based on country‐reported results from a single survey that has not undergone peer review, or even based on expert opinion alone. These estimates fitted within the same prevalence categories estimated by our study for only about 30% of all countries, highlighting the need to consider incorporating peer‐reviewed evidence in the prevalence estimation exercise for these populations. UNAIDS estimates on the prevalence in transgender people are available for only a few countries, and no information exists on MSW and TGSW.

Even though it was not possible to extrapolate the findings of MSW to country‐ and region‐specific estimates, the PR for MSW compared to MSM was higher from Kenya (respectively, 8.6 vs. 2.6) and comparable for Nigeria (respectively, 12.4 vs. 13.7), suggesting similar or higher country‐ and region HIV levels for MSW. For TGSW and transgender men, we did not find any scientific literature showing how these groups are still highly underrepresented as a key population in HIV research and programming. We highly recommend more quantitative research into the population sizes, HIV prevalence, risks, service needs and uptake for MSW, transgender women and men, and TGSW throughout SSA.

While our results do not cover access to services, limited data on access to services for MSM and transgender people suggest that access to treatment is extremely poor compared to the general population. A study that tested for antiretroviral adherence in 183 HIV‐infected MSM and transgender women in several sub‐Saharan African countries found that only 34% had antiretroviral residues in their blood, and 18% of those were not virally suppressed [80]. In addition, a systematic review by Stannah et al. [27] showed that, although HIV testing among MSM had increased significantly over the past decade, pooled estimates showed only about 23% of MSM living with HIV to be on treatment. A rough back‐of‐the‐envelope calculation using these findings and our results shows that if treatment coverage for MSM is indeed only about 25%, about 500,000–1.7 million MSM in sub‐Saharan Africa require treatment but are not receiving it. Likewise, effective pre‐exposure prophylaxis (PrEP) services rollout remains challenging. While motivation to use PrEP seems high [81], Wahome et al. found low levels of PrEP adherence and the absence of an effect on HIV incidence among MSM in SSA [79]. Despite a lack of peer‐reviewed data, it seems reasonable to assume similar or even poorer access to HIV prevention and treatment for MSW, transgender people and TGSW compared to MSM [82]. Failure to provide adequate services to these key populations could result in higher rates of morbidity, mortality and onward transmission [83]. It is essential that these services are sensitive to the unique vulnerabilities and needs of each group [9], and should coincide with minimizing structural barriers against LGBT+ people and sex workers at the personal, societal and institutional levels [9, 13, 21].

Our study has several limitations. First, we only incorporated peer‐reviewed studies. We decided not to directly include grey literature in developing our estimates due to the likely heterogeneity in quality and large risk of bias in provided estimates. Our comparison between estimates derived from peer‐reviewed literature (our review) and grey literature (UNAIDS estimates) confirms the high levels of heterogeneity between the two. Nevertheless, our peer review‐based estimates should also be cautiously interpreted in light of data limitations, selection biases and small sample sizes in the individual studies. Second, the majority of the included studies used respondent‐driven sampling (RDS) as their recruitment method. RDS has been described as the preferred sampling method for populations without a readily available sampling frame, though it has potential limitations [62, 82, 84, 85]. For example, most studies did not report on additional important indicators, such as to which extent the sample was part of the same social network [84]. People in a highly interconnected social network might not be representative of the population as a whole, as people who are not part of these networks may have different underlying characteristics and risks than those within the network. However, it is difficult to determine the direction of the potential bias, as we do not have a reliable gold standard from, for example a population‐based survey. Third, for some studies, we could not use sex‐ and age‐match general population‐level prevalence to calculate a PR, as no DHS surveys [21] were conducted within a 3‐year time window around the study. We used estimated HIV prevalence in all adults aged 15–49 years as published by Dwyer‐Lindgren et al. [25] instead. However, it is reassuring that, for areas where we could use both, reverting to PRs using data from Dwyer‐Lindgren et al. [25] did not result in any major deviations in estimated PRs (Appendix Table S16). Fourth, age standardization was often based on relatively broad age ranges (e.g. interquartile ranges) reported by the individual studies. It is likely to assume that within broad standardization categories, the age distribution among the key populations was relatively younger than the general population, resulting in an underestimation of the actual PRs. Fifth, our estimates for countries where we had no data are based on a statistical model derived from the systematic review. The sole independent predictor is HIV prevalence in the general population. These estimations should be interpreted with caution, as the regression approximation correctly predicted the data‐based prevalence categories for countries with sufficient peer‐reviewed data only 20% of the time (Appendix Tables S14 and S15). However, while we place higher confidence in estimates based on peer‐reviewed directly, it should be noted that these can also be based on relatively sparse numbers of observations, making a predicted prevalence based on pooling estimates across countries in a regression approximation not necessarily less valid. Sixth, we did not control for the year of data collection in our main analysis. Yet, we observed a borderline significant trend towards higher prevalence in later years for MSM in our sensitivity analysis (*p* = 0.04) (Appendix Table S7). This suggests that by pooling the 10 years covered by our review, we may have slightly underestimated the current PRs and HIV prevalence among MSM in sub‐Saharan Africa. However, whether this trend in time reflects an actual divergent trend in prevalence due to increased incidence and/or survival, or is caused by improved sampling approaches by which researchers are increasingly better at finding higher‐risk individuals, is difficult to determine. Seventh, all study locations were urban settings, and our PRs and estimations are, therefore, based on HIV prevalences in urban settings. We, therefore, assumed HIV prevalence for MSM and transgender women to be the same for rural settings when extrapolating our findings to national‐level estimates.

## 5 CONCLUSIONS

We show that the current HIV prevalence in MSM and transgender women in sub‐Saharan Africa is alarmingly and consistently high across all regions and countries. This high prevalence, coupled with the specific risks and vulnerabilities these populations face, highlights the urgent need for risk‐group‐tailored prevention and treatment interventions across the sub‐continent. The lack of studies in multiple countries on HIV prevalences, especially among transgender people and cisgender male and TGSWs, highlights the clear need for more research.

## COMPETING INTERESTS

The authors declare no competing interests.

## AUTHORS’ CONTRIBUTIONS

MK and JACH conceptualized and designed the study. MK, LvN and LAH performed data collection and interpretation. MK, CAB, LvN, LAH and JACH performed all analyses. MK, CAB and JACH wrote the first draft of the manuscript. All authors contributed to writing and editing the final version of the manuscript. FMC provided overall supervision.

## FUNDING

We would like to thank Aidsfonds Netherlands for funding this research project (P‐29702).

## Supporting information




**Figure S1**: Association between the HIV prevalence in the general population and in MSM.
**Figure S2**: Univariate Association between the HIV prevalence in the general population and in transgender women (TGW).
**Table S1**: Overview of complete literature searches, including updates.
**Table S2**: Bias assessment.
**Table S3**: Overview of the prevalence of HIV infection in cisgender men who have sex with men (MSM) in the included studies and prevalence of HIV in the general male population at the study location.
**Table S4**: Overview of the prevalence of HIV infection in transgender women in the included studies and prevalence of HIV in the general male and female population at the study location.
**Table S5**: Overview of the prevalence of HIV infection in cisgender male sex workers (MSW) in the included studies and prevalence of HIV in the general male population at the study location.
**Table S6**: Logistic Deming regression of the relationship between HIV prevalence in the general population and in MSM (used for extrapolation).
**Table S7**: Univariate logistic regression on relationship between HIV prevalence in MSM and potential confounders.
**Table S8**: Logistic Deming regression of the relationship between HIV prevalence in the general population and in transgender women (used for extrapolation).
**Table S9**: Univariate logistic regression on relationship between HIV prevalence in transgender women and potential confounders.
**Table S10**: Review estimations for men who have sex with men (MSM).
**Table S11**: Review estimations for transgender women (TGW).
**Table S12**: Comparison of UNAIDS estimations versus estimations from this study for men who have sex with men (MSM).
**Table S13**: Comparison of UNAIDS estimations versus estimations from this study for transgender women.
**Table S14**: Comparison of estimations derived from study data to estimations derived regression model for men who have sex with men (MSM).
**Table S15**: Comparison of estimations derived from study data to estimations derived regression model for transgender women.
**Table S16**: Assesment of the impact of the use of geospatially matched Demographic Health Surveys (DHS) (52) data versus Dwyer‐Lindgren et al. (56) (DL) data.

## Data Availability

Data sharing is not applicable to this article as no new data were created or analysed in this study.
