# Supplementary material for: HIV prevalence among men who have sex with men, transgender women and cisgender male sex workers in sub‐Saharan Africa: a systematic review and meta‐analysis
Source: J Int AIDS Soc. 2022 Nov 23;25(11):e26022. doi: 10.1002/jia2.26022 (PMC9684687; doi:10.1002/jia2.26022)
Supplement: Supplementary file 1 — Figure S1: Association between the HIV prevalence in the general population and in MSM. Figure S2: Univariate Association between the HIV prevalence in the general population and in transgender women (TGW). Table S1: Overview of complete literature searches, including updates. Table S2: Bias assessment. Table S3: Overview of the prevalence of HIV infection in cisgender men who have sex with men (MSM) in the included studies and prevalence of HIV in the general male population at the study location. Table S4: Overview of the prevalence of HIV infection in transgender women in the included studies and prevalence of HIV in the general male and female population at the study location. Table S5: Overview of the prevalence of HIV infection in cisgender male sex workers (MSW) in the included studies and prevalence of HIV in the general male population at the study location. Table S6: Logistic Deming regression of the relationship between HIV prevalence in the general population and in MSM (used for extrapolation). Table S7: Univariate logistic regression on relationship between HIV prevalence in MSM and potential confounders. Table S8: Logistic Deming regression of the relationship between HIV prevalence in the general population and in transgender women (used for extrapolation). Table S9: Univariate logistic regression on relationship between HIV prevalence in transgender women and potential confounders. Table S10: Review estimations for men who have sex with men (MSM). Table S11: Review estimations for transgender women (TGW). Table S12: Comparison of UNAIDS estimations versus estimations from this study for men who have sex with men (MSM). Table S13: Comparison of UNAIDS estimations versus estimations from this study for transgender women. Table S14: Comparison of estimations derived from study data to estimations derived regression model for men who have sex with men (MSM). Table S15: Comparison of estimations derived from study data to estimations derived regression m [file JIA2-25-e26022-s001.docx]

# **Supplementary appendix to:**

**HIV prevalence among men who have sex with men, transgender women, and cisgender male sex workers in sub-Saharan Africa: a systematic review and meta-analysis**

**File S1: Systematic literature search**

**Date of search**

4^th^ June 2018

**Question**

What is the population size estimate cq. HIV prevalence and risk in sex workers or men who have sex with men (MSM) in sub-Saharan African countries?

**Search results**

| ***Database*** | ***Number of refs*** | ***Refs after deduplication*** |
| --- | --- | --- |
|  |  |  |
| Embase.com | 1,426 | 1,400 |
| Medline Epub (Ovid) | 1,506 | 387 |
|  |  |  |
| Web of Science | 1,788 | 831 |
| Google Scholar | 200 | 166 |
|  |  |  |
| **Total** | **4,920** | **2,784** |

*Deduplicates: 2,136*

**Search terms**

***Embase.com (embase incl. Medline): 1,426***

**(**'LGBT people'/exp *OR 'sex worker'/de* OR (LGBT* OR GLBT* OR transgender* *OR transsexual* OR MSM* *OR sexwork** *OR prostitute** OR ((sex OR escort) NEAR/4 (*work* OR sell* OR money OR gift* OR surviv** *OR transaction** OR men OR man)) OR ((money OR call) NEAR/3 (boy*)) OR *gay* OR ((cross) NEXT/1 (dress*)) OR ((drag) NEXT/1 (queen*)) OR genderqueer* OR ((gender) NEXT/1 (queer OR nonconforming)) *OR homosexual* OR bisexual**):ab,ti**) AND (**'Human immunodeficiency virus infection'/exp OR (HIV OR AIDS OR ((human) NEXT/1 (immunodeficiency) NEXT/1 (virus))):ab,ti**) AND (**'prevalence'/exp *OR 'cross-sectional study'/de* OR 'incidence'/exp OR 'odds ratio'/de *OR 'risk factor'/de* OR (Prevalen* *OR "cross-sectional"* OR incidence* *OR ((risk* OR odds) NEAR/1 (factor* OR relative OR ratio*))*):ab,ti**) AND (**'Africa south of the Sahara'/exp OR (((black OR Central OR South) NEAR/3 (Africa*)) OR *'sub-Saharan' OR* subSaharan* OR Angola OR Benin OR Botswana OR 'Burkina Faso' OR Burundi OR Cameroon OR 'Cape Verde' OR Chad OR Comoro* OR Congo *OR ((Cote) NEAR/3 (Ivoir*))* OR Djibouti OR Eritrea OR Ethiopia OR Gabon OR Gambia OR Ghana OR Guinea OR Kenya OR Lesotho OR Liberia OR Madagascar OR Malawi OR Mali OR Mayotte OR Mozambique OR Namibia OR Niger OR Nigeria OR Rwanda OR Sahel OR Senegal OR 'Sierra Leone' OR Somalia OR Sudan OR Swaziland OR Tanzania OR Togo OR Uganda OR Zambia OR Zimbabwe):ab,ti**) NOT (**'Conference Abstract' OR Editorial**)/**it

***Medline Epub: 1,506***

**(**exp "Sexual and Gender Minorities"/ *OR "Sex Workers"/* OR (LGBT* OR GLBT* OR transgender* *OR transsexual* OR MSM* *OR sexwork** *OR prostitute** OR ((sex OR escort) ADJ4 (*work* OR sell* OR money OR gift* OR surviv** *OR transaction** OR men OR man)) OR ((money OR call) ADJ3 (boy*)) OR *gay* OR ((cross) ADJ1 (dress*)) OR ((drag) ADJ1 (queen*)) OR genderqueer* OR ((gender) ADJ1 (queer OR nonconforming)) *OR homosexual* OR bisexual**).ab,ti.**) AND (**exp HIV/ OR (HIV OR AIDS OR ((human) ADJ1 (immunodeficiency) *ADJ1* (virus))).ab,ti.**) AND (**Prevalence/ *OR Cross-Sectional Studies/* OR Incidence/ OR Odds Ratio/ *OR Risk Factors/* OR (prevalen* *OR "cross-sectional"* OR incidence* *OR ((risk OR odds) ADJ1 (factor* OR relative OR ratio*))*).ab,ti.**) AND (**Africa south of the Sahara/ OR (((black OR Central OR South) ADJ3 (Africa*)) OR "*sub-Saharan" OR* subSaharan* OR Angola OR Benin OR Botswana OR Burkina Faso OR Burundi OR Cameroon OR Cape Verde OR Chad OR Comoro* OR Congo *OR ((Cote) ADJ3 (Ivoir*))* OR Djibouti OR Eritrea OR Ethiopia OR Gabon OR Gambia OR Ghana OR Guinea OR Kenya OR Lesotho OR Liberia OR Madagascar OR Malawi OR Mali OR Mayotte OR Mozambique OR Namibia OR Niger OR Nigeria OR Rwanda OR Sahel OR Senegal OR Sierra Leone OR Somalia OR Sudan OR Swaziland OR Tanzania OR Togo OR Uganda OR Zambia OR Zimbabwe).ab,ti.**) NOT (c**ongresses OR editorial**)**.pt.

***Web of Science: 1,788***

**TS=((**(LGBT* OR GLBT* OR transgender* *OR transsexual* OR MSM* *OR sexwork** *OR prostitute** OR ((sex OR escort) NEAR/4 (*work* OR sell* OR money OR gift* OR surviv** *OR transaction** OR men OR man)) OR ((money OR call) NEAR/2 (boy*)) OR *gay* OR ((cross) NEAR/1 (dress*)) OR ((drag) NEAR/1 (queen*)) OR genderqueer* OR ((gender) NEAR/1 (queer OR nonconforming)) *OR homosexual* OR bisexual**)**) AND (**(HIV OR AIDS OR ((human) NEAR/1 (immunodeficiency) NEAR/1 (virus)))**) AND (**(Prevalen* *OR "cross-sectional"* OR incidence* *OR ((risk* OR odds) NEAR/1 (factor* OR relative OR ratio*))*)**) AND (**(((black OR Central OR South) NEAR/2 (Africa*)) OR "*sub-Saharan" OR* subSaharan* OR Angola OR Benin OR Botswana OR "Burkina Faso" OR Burundi OR Cameroon OR "Cape Verde" OR Chad OR Comoro* OR Congo *OR ((Cote) NEAR/2 (Ivoir*))* OR Djibouti OR Eritrea OR Ethiopia OR Gabon OR Gambia OR Ghana OR Guinea OR Kenya OR Lesotho OR Liberia OR Madagascar OR Malawi OR Mali OR Mayotte OR Mozambique OR Namibia OR Niger OR Nigeria OR Rwanda OR Sahel OR Senegal OR "Sierra Leone" OR Somalia OR Sudan OR Swaziland OR Tanzania OR Togo OR Uganda OR Zambia OR Zimbabwe)**)**) **AND** DT=Article

***Google Scholar: 200*** *(top relevant refs)*

LGBT|GLBT|transgender|*transsexual|MSM*|*prostitute|*"sex|escort *worker"|gay*|*homosexual|bisexual* HIV|AIDS|"human immunodeficiency virus" prevalence|*"cross-sectional"|*incidence|*"risk|odds factor|ratio|"relative risk***" "***sub-Saharan"|*"black|central Africa"

**Date of updated search 2020**

28 July 2020

**Search results**

| ***Database*** | ***Number of refs*** | ***Refs after deduplication*** |
| --- | --- | --- |
|  |  |  |
| Embase.com | 1780 | 1754 |
| Medline Epub (Ovid) | 1820 | 383 |
|  |  |  |
| Web of Science | 2089 | 816 |
| Google Scholar | 200 | 168 |
|  |  |  |
| **Total** | **5859** | **3121** |

*Deduplicates: 2,738*

*New references (in EndNote): 671*

**Search terms**

***Embase.com (embase incl. Medline):***

**(**'LGBT people'/exp *OR 'sex worker'/de* OR (LGBT* OR GLBT* OR transgender* *OR transsexual* OR MSM* *OR sexwork** *OR prostitute** OR ((sex OR escort) NEAR/4 (*work* OR sell* OR money OR gift* OR surviv** *OR transaction** OR men OR man)) OR ((money OR call) NEAR/3 (boy*)) OR *gay* OR ((cross) NEXT/1 (dress*)) OR ((drag) NEXT/1 (queen*)) OR genderqueer* OR ((gender) NEXT/1 (queer OR nonconforming)) *OR homosexual* OR bisexual**):ab,ti**) AND (**'Human immunodeficiency virus infection'/exp OR (HIV OR AIDS OR ((human) NEXT/1 (immunodeficiency) NEXT/1 (virus))):ab,ti**) AND (**'prevalence'/exp *OR 'cross-sectional study'/de* OR 'incidence'/exp OR 'odds ratio'/de *OR 'risk factor'/de* OR (Prevalen* *OR "cross-sectional"* OR incidence* *OR ((risk* OR odds) NEAR/1 (factor* OR relative OR ratio*))*):ab,ti**) AND (**'Africa south of the Sahara'/exp OR (((black OR Central OR South) NEAR/3 (Africa*)) OR *'sub-Saharan' OR* subSaharan* OR Angola OR Benin OR Botswana OR 'Burkina Faso' OR Burundi OR Cameroon OR 'Cape Verde' OR Chad OR Comoro* OR Congo *OR ((Cote) NEAR/3 (Ivoir*))* OR Djibouti OR Eritrea OR Ethiopia OR Gabon OR Gambia OR Ghana OR Guinea OR Kenya OR Lesotho OR Liberia OR Madagascar OR Malawi OR Mali OR Mayotte OR Mozambique OR Namibia OR Niger OR Nigeria OR Rwanda OR Sahel OR Senegal OR 'Sierra Leone' OR Somalia OR Sudan OR Swaziland OR Tanzania OR Togo OR Uganda OR Zambia OR Zimbabwe):ab,ti**) NOT (**'Conference Abstract' OR Editorial**)/**it

***Medline Epub:***

**(**exp "Sexual and Gender Minorities"/ *OR "Sex Workers"/* OR (LGBT* OR GLBT* OR transgender* *OR transsexual* OR MSM* *OR sexwork** *OR prostitute** OR ((sex OR escort) ADJ4 (*work* OR sell* OR money OR gift* OR surviv** *OR transaction** OR men OR man)) OR ((money OR call) ADJ3 (boy*)) OR *gay* OR ((cross) ADJ1 (dress*)) OR ((drag) ADJ1 (queen*)) OR genderqueer* OR ((gender) ADJ1 (queer OR nonconforming)) *OR homosexual* OR bisexual**).ab,ti.**) AND (**exp HIV/ OR (HIV OR AIDS OR ((human) ADJ1 (immunodeficiency) *ADJ1* (virus))).ab,ti.**) AND (**Prevalence/ *OR Cross-Sectional Studies/* OR Incidence/ OR Odds Ratio/ *OR Risk Factors/* OR (prevalen* *OR "cross-sectional"* OR incidence* *OR ((risk OR odds) ADJ1 (factor* OR relative OR ratio*))*).ab,ti.**) AND (**Africa south of the Sahara/ OR (((black OR Central OR South) ADJ3 (Africa*)) OR "*sub-Saharan" OR* subSaharan* OR Angola OR Benin OR Botswana OR Burkina Faso OR Burundi OR Cameroon OR Cape Verde OR Chad OR Comoro* OR Congo *OR ((Cote) ADJ3 (Ivoir*))* OR Djibouti OR Eritrea OR Ethiopia OR Gabon OR Gambia OR Ghana OR Guinea OR Kenya OR Lesotho OR Liberia OR Madagascar OR Malawi OR Mali OR Mayotte OR Mozambique OR Namibia OR Niger OR Nigeria OR Rwanda OR Sahel OR Senegal OR Sierra Leone OR Somalia OR Sudan OR Swaziland OR Tanzania OR Togo OR Uganda OR Zambia OR Zimbabwe).ab,ti.**) NOT (c**ongresses OR editorial**)**.pt.

***Web of Science:***

**TS=((**(LGBT* OR GLBT* OR transgender* *OR transsexual* OR MSM* *OR sexwork** *OR prostitute** OR ((sex OR escort) NEAR/4 (*work* OR sell* OR money OR gift* OR surviv** *OR transaction** OR men OR man)) OR ((money OR call) NEAR/2 (boy*)) OR *gay* OR ((cross) NEAR/1 (dress*)) OR ((drag) NEAR/1 (queen*)) OR genderqueer* OR ((gender) NEAR/1 (queer OR nonconforming)) *OR homosexual* OR bisexual**)**) AND (**(HIV OR AIDS OR ((human) NEAR/1 (immunodeficiency) NEAR/1 (virus)))**) AND (**(Prevalen* *OR "cross-sectional"* OR incidence* *OR ((risk* OR odds) NEAR/1 (factor* OR relative OR ratio*))*)**) AND (**(((black OR Central OR South) NEAR/2 (Africa*)) OR "*sub-Saharan" OR* subSaharan* OR Angola OR Benin OR Botswana OR "Burkina Faso" OR Burundi OR Cameroon OR "Cape Verde" OR Chad OR Comoro* OR Congo *OR ((Cote) NEAR/2 (Ivoir*))* OR Djibouti OR Eritrea OR Ethiopia OR Gabon OR Gambia OR Ghana OR Guinea OR Kenya OR Lesotho OR Liberia OR Madagascar OR Malawi OR Mali OR Mayotte OR Mozambique OR Namibia OR Niger OR Nigeria OR Rwanda OR Sahel OR Senegal OR "Sierra Leone" OR Somalia OR Sudan OR Swaziland OR Tanzania OR Togo OR Uganda OR Zambia OR Zimbabwe)**)**) **AND** DT=Article

***Google Scholar: 200*** *(top relevant refs)*

LGBT|GLBT|transgender|*transsexual|MSM*|*prostitute|*"sex|escort *worker"|gay*|*homosexual|bisexual* HIV|AIDS|"human immunodeficiency virus" prevalence|*"cross-sectional"|*incidence|*"risk|odds factor|ratio|relative***" "***sub-Saharan"|*"black|central Africa"

**Date of updated search 2021**

22-10-2021

**Search results**

| ***Database*** | ***Number of refs*** | ***Refs after deduplication*** |
| --- | --- | --- |
|  |  |  |
| Embase.com | 2068 | 2041 |
| Medline (Ovid) | 2098 | 418 |
| Africa Index Medicus | 44 | 31 |
| Web of Science | 2453 | 903 |
| Google Scholar (200 top-ranked) | 200 | 128 |
|  |  |  |
| **Total** | **6863** | **3521** |

*Deduplicates: 3,342*

*Already included in initial search with first update: 2,924*

*New references (in EndNote): 597*

**Manual search**

| ***Database*** | ***Number of refs*** |
| --- | --- |
|  |  |
| Africa Journals Online | 161 |
|  |  |
| **Total** | **161** |

**Search terms**

***Embase***

*('LGBT people'/exp OR 'sex worker'/de OR (LGBT* OR GLBT* OR transgender* OR transsexual* OR MSM OR sexwork* OR prostitute* OR ((sex OR escort) NEAR/4 (work* OR sell* OR money OR gift* OR surviv* OR transaction* OR men OR man)) OR ((money OR call) NEAR/3 (boy*)) OR gay OR ((cross) NEXT/1 (dress*)) OR ((drag) NEXT/1 (queen*)) OR genderqueer* OR ((gender) NEXT/1 (queer OR nonconforming)) OR homosexual* OR bisexual*):ab,ti,kw) AND ('Human immunodeficiency virus infection'/exp OR (HIV OR AIDS OR ((human) NEXT/1 (immunodeficiency) NEXT/1 (virus))):ab,ti,kw) AND ('prevalence'/exp OR 'cross-sectional study'/de OR 'incidence'/exp OR 'odds ratio'/de OR 'risk factor'/de OR (Prevalen* OR "cross-sectional" OR incidence* OR ((risk* OR odds) NEAR/1 (factor* OR relative OR ratio*))):ab,ti,kw) AND ('Africa south of the Sahara'/exp OR (((black OR Central OR South) NEAR/3 (Africa*)) OR 'sub-Saharan' OR subSaharan* OR Angola OR Benin OR Botswana OR 'Burkina Faso' OR Burundi OR Cameroon OR 'Cape Verde' OR Chad OR Comoro* OR Congo OR ((Cote) NEAR/3 (Ivoir*)) OR Djibouti OR Eritrea OR Ethiopia OR Gabon OR Gambia OR Ghana OR Guinea OR Kenya OR Lesotho OR Liberia OR Madagascar OR Malawi OR Mali OR Mayotte OR Mozambique OR Namibia OR Niger OR Nigeria OR Rwanda OR Sahel OR Senegal OR 'Sierra Leone' OR Somalia OR Sudan OR Swaziland OR Tanzania OR Togo OR Uganda OR Zambia OR Zimbabwe):ab,ti,kw) NOT ('Conference Abstract' OR Editorial)/it*

***Medline***

*(exp "Sexual and Gender Minorities"/ OR "Sex Workers"/ OR (LGBT* OR GLBT* OR transgender* OR transsexual* OR MSM OR sexwork* OR prostitute* OR ((sex OR escort) ADJ4 (work* OR sell* OR money OR gift* OR surviv* OR transaction* OR men OR man)) OR ((money OR call) ADJ3 (boy*)) OR gay OR ((cross) ADJ1 (dress*)) OR ((drag) ADJ1 (queen*)) OR genderqueer* OR ((gender) ADJ1 (queer OR nonconforming)) OR homosexual* OR bisexual*).ab,ti,kf.) AND (exp HIV/ OR (HIV OR AIDS OR ((human) ADJ1 (immunodeficiency) ADJ1 (virus))).ab,ti,kf.) AND (Prevalence/ OR Cross-Sectional Studies/ OR Incidence/ OR Odds Ratio/ OR Risk Factors/ OR (prevalen* OR "cross-sectional" OR incidence* OR ((risk OR odds) ADJ1 (factor* OR relative OR ratio*))).ab,ti,kf.) AND (Africa south of the Sahara/ OR (((black OR Central OR South) ADJ3 (Africa*)) OR "sub-Saharan" OR subSaharan* OR Angola OR Benin OR Botswana OR Burkina Faso OR Burundi OR Cameroon OR Cape Verde OR Chad OR Comoro* OR Congo OR ((Cote) ADJ3 (Ivoir*)) OR Djibouti OR Eritrea OR Ethiopia OR Gabon OR Gambia OR Ghana OR Guinea OR Kenya OR Lesotho OR Liberia OR Madagascar OR Malawi OR Mali OR Mayotte OR Mozambique OR Namibia OR Niger OR Nigeria OR Rwanda OR Sahel OR Senegal OR Sierra Leone OR Somalia OR Sudan OR Swaziland OR Tanzania OR Togo OR Uganda OR Zambia OR Zimbabwe).ab,ti,kf.) NOT (news OR congres* OR abstract* OR book* OR chapter* OR dissertation abstract* OR editorial).pt.*

***Web of Science:***

*TS=(((LGBT* OR GLBT* OR transgender* OR transsexual* OR MSM OR sexwork* OR prostitute* OR ((sex OR escort) NEAR/4 (work* OR sell* OR money OR gift* OR surviv* OR transaction* OR men OR man)) OR ((money OR call) NEAR/2 (boy*)) OR gay OR ((cross) NEAR/1 (dress*)) OR ((drag) NEAR/1 (queen*)) OR genderqueer* OR ((gender) NEAR/1 (queer OR nonconforming)) OR homosexual* OR bisexual*)) AND ((HIV OR AIDS OR ((human) NEAR/1 (immunodeficiency) NEAR/1 (virus)))) AND ((Prevalen* OR "cross-sectional" OR incidence* OR ((risk* OR odds) NEAR/1 (factor* OR relative OR ratio*)))) AND ((((black OR Central OR South) NEAR/2 (Africa*)) OR "sub-Saharan" OR subSaharan* OR Angola OR Benin OR Botswana OR "Burkina Faso" OR Burundi OR Cameroon OR "Cape Verde" OR Chad OR Comoro* OR Congo OR ((Cote) NEAR/2 (Ivoir*)) OR Djibouti OR Eritrea OR Ethiopia OR Gabon OR Gambia OR Ghana OR Guinea OR Kenya OR Lesotho OR Liberia OR Madagascar OR Malawi OR Mali OR Mayotte OR Mozambique OR Namibia OR Niger OR Nigeria OR Rwanda OR Sahel OR Senegal OR "Sierra Leone" OR Somalia OR Sudan OR Swaziland OR Tanzania OR Togo OR Uganda OR Zambia OR Zimbabwe))) AND DT=(Article OR Review OR Letter OR Early Access)*

***Google Scholar: 200 (top relevant refs)***

*LGBT|GLBT|transgender|transsexual|MSM|prostitute|"sex|escort worker"|gay|homosexual|bisexual HIV|AIDS|"human immunodeficiency virus" prevalence|"cross-sectional"|incidence|"risk|odds factor|ratio|relative" "sub-Saharan"|"black|central Africa"*

*LGBT|GLBT|transgender|transsexual|MSM|prostitute|'sex|escort worker'|gay|homosexual|bisexual HIV|AIDS|'human immunodeficiency virus' prevalence|'cross-sectional'|incidence|'risk|odds factor|ratio|relative' 'sub-Saharan'|'black|central Africa'*

***Africa Index Medicus (via*** [***www.globalindexmedicus.net***](http://www.globalindexmedicus.net)***) 44***

*(mh:("Sex Workers" OR "Sexual and Gender Minorities") OR TW:(LGBT* OR GLBT* OR transgender* OR transsexual* OR MSM OR sexwork* OR prostitute* OR escort* OR "money boy" OR "call boy" OR gay* OR "cross dress" OR "drag queen" OR genderqueer* OR nonconforming* OR homosexual* OR bisexual*)) AND (mh:("HIV Infections") OR TW:(HIV OR AIDS OR "immunodeficiency virus"))*

***Africa Journals Online 161***

*Journal database is manually searched, because database doesn't allow to export results: manually entered in the search box (1) and then manually screened for new articles. 161 results on 22-10-2021*

*(LGBT* OR GLBT* OR transgender* OR transsexual* OR MSM OR sexwork* OR prostitute* OR escort* OR money-boy OR call-boy OR gay* OR cross-dress* OR drag-queen* OR genderqueer* OR nonconforming* OR homosexual* OR bisexual*) AND (HIV OR AIDS OR immunodeficiency-virus)*

**Table S1. Overview of complete literature searches, including updates**

|  | **Search 2020** | **Search 2020 with new in-/exclusion criteria 2021** | | **Update 2021** | | **Manual search African Journal Online 2021** | |
| --- | --- | --- | --- | --- | --- | --- | --- |
|  | **Count** | ***Number of added/deleted studies*** | **Total Count 2020** | ***Number of studies from update 2021*** | **Total Count 2021** | ***Number of studies from manual search 2021*** | **Final Count 2021** |
| **Eligible studies** | **5376** |  | **5376** | ***6863*** | **9315** | ***161*** | **9476** |
| Excluded | 3420 |  | 3420 | *6393* | 6889 | *0* | 6889 |
| Duplicates | 2152 |  | 2152 | *3342* | 5494 |  | 5494 |
| Published before 2010 | 1268 |  | 1268 | *127* | 1395 |  | 1395 |
| Already included in 2020 search |  |  |  | *2924* |  |  |  |
| **Included for review title and abstract** | **1956** |  | **1956** | ***470*** | **2426** | ***161*** | **2587** |
| Excluded | 1782 |  | 1782 | *438* | 2220 | *160* | 2380 |
| **Included for review full text** | **174** |  | **174** | ***32*** | **206** | ***1*** | **207** |
| Excluded | 125 | *12* | 137 | *21* | 158 | *1* | 159 |
| **Final included** | **49** | ***37*** | **37** | ***11*** | **48** | ***0*** | **48** |
| Cisgender men who have sex with men | 44 | *-11* | 33 | *11* | 44 | *0* | 44 |
| Transgender women | 4 | *0* | 4 | *6* | 10 | *0* | 10 |
| Cisgender male sex workers | 5 | *0* | 5 | *0* | 5 | *0* | 5 |
| Transgender men | 0 | *0* | 0 | *0* | 0 | *0* | 0 |
| Transgender sex workers | 0 | *0* | 0 | *0* | 0 | *0* | 0 |

**Panel S1: Terminology; Men who have sex with men (MSM), transgender men and women and cisgender male and transgender sex workers (MSW and TGSW).**

The terminology we used for the classifications and definitions of our study groups are derived from ILGA Europe(2), and consists of four concepts; sex, gender identity, sexual orientation, and type of sex partners. Sex is the biological sex assigned at birth (male or female). Gender identity refers to the combination of sex and gender of a person (how a person feels, e.g. man, woman, or other), and can be cisgender (gender and sex at birth are typically aligned) or transgender (gender and sex at birth are not typically aligned), and is independent of gender affirming treatment or surgery. Sexual orientation describes a person’s capacity for profound affection, emotional and sexual attraction to, and intimate and sexual relations with, individuals of a different gender or the same gender or more than one gender and the most common orientations are heterosexual (other gender), homosexual (same gender), bisexual (more than one gender). Queer is an umbrella term used to express a spectrum of gender identities and sexual orientations beyond the binary. Type of sex partners is about sexual behavior and describes who a person has sex with, which does not have to be limited by sexual orientation. Besides sex assigned at birth, all concepts can naturally fluctuate over time on a personal level. In our study, we classified men who have sex with men (MSM) as cisgender men who have men sex partners, but may have other type of sex partners as well. Transgender men are people who identify as men and were female sex assigned at birth, and transgender women are people who identify as women and were male sex assigned at birth. Being transgender does not define a person’s sexual orientation or sexual behavior. Transgender men and -women can work as sex worker (transgender sex workers (TGSW)), and cisgender men can work as sex worker (cisgender male sex workers), regardless of their sexual orientation and type of sex partners. We here refer to “male” sex workers rather than “men” as this is the commonly accepted term for this group.

We have only included studies in this review based on these terms. For example, we assessed the HIV prevalence among the full spectrum of MSW, or cisgender men sex workers, meaning; male sex assigned at birth, identifying as man and offering sex. These MSW can have various sexual orientations (for example: heterosexual, homosexual or bisexual) and various types of clients (male, female or both). MSW in sub-population of MSM would therefore not be a representative sample of all MSW, and it is not possible assess to which extend and in which direction the HIV prevalence is influenced by the fact that a study only included MSW who are MSM. Future research should focus on clearly and appropriately defining the groups.

**Table S2: Bias assessment.** Bias amongst the included studies was assessed using the Joanna Briggs Institute (JBI) critical appraisal checklist for studies reporting prevalence data(3). Answer options are Y = Yes or N = No.

| **Study** | **Was the sample frame appropriate to address the target population?** | **Were study participant sampled in an appropriate way?** | **Was the sample size adequate?** | **Were study subjects and the setting described in detail?** | **Was the data analysis conducted with sufficient coverage of the identified sample?** | **Were valid methods used for the identification of the condition?** | **Was the condition measured in a standard, reliable way for all participants?** | **Was there appropriate statistical analysis?** | **Was the response rate adequate, and if not, was the low response rate managed appropriate?** |
| --- | --- | --- | --- | --- | --- | --- | --- | --- | --- |
| Kendall *et al.* (2014)(3) | Y | Y | Y | Y | Y | Y | Y | Y | Y |
| Tafuma *et al.* (2014)(4) | Y | Y | Y | Y | Y | Y | Y | Y | Y |
| Holland *et al.* (2016)(5) | Y | Y | Y | Y | Y | Y | Y | Y | Y |
| Yaya *et al.* (2021)(6) | Y | Y | Y | Y | Y | Y | Y | Y | Y |
| Park *et al.* (2013)(7) | Y | Y | Y | Y | Y | Y | Y | Y | Y |
| Marcel *et al.* (2019)(8) | Y | Y | Y | Y | Y | Y | Y | Y | Y |
| Hakim *et al.* (2015)(9) | Y | Y | Y | Y | Y | Y | Y | Y | Y |
| Mason *et al.* (2013)(10) | Y | Y | Y | Y | Y | Y | Y | Y | Y |
| Muraguri *et al.* (2015)(11) | Y | Y | Y | Y | Y | Y | Y | Y | Y |
| Battacharjee *et al.* (2020)(12) | Y | Y | Y | Y | Y | Y | Y | Y | Y |
| Gebrebrhan *et al.* (2020)(13) | Y | Y | Y | Y | Y | Y | Y | Y | Y |
| Sandfort *et al.* (2019)(15) | Y | Y | Y | Y | Y | Y | Y | Y | Y |
| Smith *et al.* (2021)(15) | Y | Y | Y | Y | Y | Y | Y | Y | Y |
| Stahlman *et al.* (2016)(16) | Y | Y | Y | Y | Y | Y | Y | Y | Y |
| Wirtz *et al.* (2017)(17) | Y | Y | Y | Y | Y | Y | Y | Y | Y |
| Lahuerta *et al.* (2017)(19) | Y | Y | Y | Y | Y | Y | Y | Y | Y |
| Kayolta *et al.* (2021)(19) | Y | Y | Y | Y | Y | Y | Y | Y | Y |
| Nalá *et al.* (2015)(20) | Y | Y | Y | Y | Y | Y | Y | Y | Y |
| Keshinro *et al.* (2016)(21) | Y | Y | Y | Y | Y | Y | Y | Y | Y |
| Bamgboye *et al.* (2017)(23) | Y | Y | Y | Y | Y | Y | Y | Y | Y |
| Vu *et al.* (2013)(23) | Y | Y | Y | Y | Y | Y | Y | Y | Y |
| Ramadhani *et al.* (2020)(24) | Y | Y | Y | Y | Y | Y | Y | Y | Y |
| Ntale *et al.* (2018)(26) | Y | Y | Y | Y | Y | Y | Y | Y | Y |
| Murunzi *et al.* (2020)(27) | Y | Y | Y | Y | Y | Y | Y | Y | Y |
| Twahirwa Rwema *et al.* (2020)(28) | Y | Y | Y | Y | Y | Y | Y | Y | Y |
| Dramé *et al.* (2013)(29) | Y | Y | Y | Y | Y | Y | Y | Y | Y |
| Lyons *et al.* (2017)(29) | Y | Y | Y | Y | Y | Y | Y | Y | Y |
| Jobson *et al.* (2018)(30) | Y | Y | Y | Y | Y | Y | Y | Y | Y |
| Fearon *et al.* (2017)(32) | Y | Y | Y | Y | Y | Y | Y | Y | Y |
| Lane *et al.* (2014)(32) | Y | Y | Y | Y | Y | Y | Y | Y | Y |
| Sandfort *et al.* (2015)(33) | Y | Y | Y | Y | Y | Y | Y | Y | Y |
| Sullivan *et al.* (2020)(34) | Y | Y | Y | Y | Y | Y | Y | Y | Y |
| Baral *et al.* (2013)(36) | Y | Y | Y | Y | Y | Y | Y | Y | Y |
| Ross *et al.* (2014)(36) | Y | Y | Y | Y | Y | Y | Y | Y | Y |
| Khatib *et al.* (2017)(37) | Y | Y | Y | Y | Y | Y | Y | Y | Y |
| Ishungisa *et al.* (2020)(38) | Y | Y | Y | Y | Y | Y | Y | Y | Y |
| Mmbaga *et al*. (2017)(39) | Y | Y | Y | Y | Y | Y | Y | Y | Y |
| Mmbaga *et al.* (2018)(41) | Y | Y | Y | Y | Y | Y | Y | Y | Y |
| Teclessou *et al.* (2017)(41) | Y | Y | Y | Y | Y | Y | Y | Y | Y |
| Tchankoni *et al.* (2020)(42) | Y | Y | Y | Y | Y | Y | Y | Y | Y |
| Ferré *et al.* (2019)(44) | Y | Y | Y | Y | Y | Y | Y | Y | Y |
| Hladik *et al.* (2017)(44) | Y | Y | Y | Y | Y | Y | Y | Y | Y |
| Parmley *et al.* (2021)(45) | Y | Y | Y | Y | Y | Y | Y | Y | Y |
| Poteat *et al*. (2017)(46) | Y | Y | Y | Y | Y | Y | Y | Y | Y |
| Stahlman *et al.* (2016)(47) | Y | Y | Y | Y | Y | Y | Y | Y | Y |
| McKinnon *et al.* (2014)(48) | Y | Y | Y | Y | Y | Y | Y | Y | Y |
| Smith *et al.* (2015)(49) | Y | Y | Y | Y | Y | Y | Y | Y | Y |
| Crowell *et al.* (2016)(50) | Y | Y | Y | Y | Y | Y | Y | Y | Y |

All bias indicators were assessed based on the purpose of the data in our study and most indicators are part of the exclusion criteria. We graded Yes for all studies if the type of *sample frame* met our in-/exclusion criteria. Therefore, all included studies are graded Yes. Likewise, for whether *participants were sampled in an appropriate way*. If for example targeting a specific high risk MSM group, or subgroup amongst MSM (such as drug using MSM) we excluded the study. Regarding the *sample size* and whether the *data analysis conducted with sufficient coverage of the identified sample*, for the forestplots (fig 3-6), country- and region prevalence and prevalence ratios were calculated weighted by study size. Therefore, a minimum sample size was not needed. However, for the maps with estimated country prevalence (fig 7) we excluded studies with a sample size n<80 from the statistical model, and the statistical model was used for countries with no data or a country prevalence based on studies with a total of n<80. For the question whether *subjects and setting were described in detail*, for us it was important to know how MSM, transgender women and MSW were identified in the study. In some cases we would find misclassification based on inappropriate classification of these groups (as described in panel S1 terminology). If aggregated data was not presented, we have contacted authors and retrieved aggregated data. *Setting details* were needed to retrieve the location of the study. Then, *were valid methods used for the identification of the condition,* and *was the condition measured in a standard, reliable way for all participants*?, were fully answered with “yes”, as we only included studies that report results based on laboratory HIV testing of blood. We excluded studies with self-testing only, or based on self-reported HIV status. *Was there appropriate statistical analysis?* For each study, we checked the prevalence calculation based on the total number of people tested and number of people infected. Inconsistencies were discussed with the corresponding authors. Last, *was the response rate adequate;* this question is very difficult to answer, as little is known about the population size of these communities and many people are not linked to HIV services and study outreach activities due to access barriers. Overall, we graded all studies based on the JBI critical appraisal checklist of sufficient quality.

**Table S3: Overview of the prevalence of HIV infection in cisgender men who have sex with men (MSM) in the included studies and prevalence of HIV in the general male population at the study location.**

| **Study** | **Country and location** | **Year(s) of study recruitment** | **Recruitment method** | **No. tested for HIV** | **Mean or median and IQR or range of age of the total population** | **HIV prevalence (%)**  **(95% confidence interval)** | **HIV prevalence general male population in study recruitment year (%) (95% confidence interval)** |
| --- | --- | --- | --- | --- | --- | --- | --- |
|  | Angola |  |  |  |  |  |  |
| Kendall *et al.* (2014)(3) | Luanda Province | 2011 | RDS | 328 | > 18 | 3.7 (1.5-6.3) ^c^ | 0.9 (0.5–1.2) ͙ͩ |
|  | Botswana |  |  |  |  |  |  |
| Tafuma *et al.* (2014)(4) | Gaborone | 2012 | RDS | 275 | 23.2 (18–53) | 12.3 (8.4–16.3) ^a^ | 14.2 (14.2–14.2) |
|  | Francistown |  |  | 145 |  | 11.7 (6.5–16.9) ^a^ | 20.2 (20.1–20.2) |
|  | Kasane |  |  | 30 |  | 25.9 (9.0–42.8) ^a^ | 17.7 (17.4–18.0) |
|  | Burkina Faso |  |  |  |  |  |  |
| Holland *et al.* (2016)(5) | Ouagadougou | 2013 | RDS | 339 | > 18 | 4.7 (2.8–7.7) ^a^ | 1.3 (1.0–1.6) ͙ͩ |
|  | Bobo Dioulasso |  |  | 329 |  | 4.9 (2.9–8.0) ^a^ | 2.0 (1.5–2.5) ͙ͩ |
| Yaya *et al.* (2021)(6) | Ouagadougou | 2017-2018 | RDS | 144 | Agegroup 18-24  40.1%,  Agegroup 25-34  51.0%,  Agegroup 35+  8.9% | 31.9 (24.3-39.6) ^a^ | 0.9 (0.0-2.0) |
|  | Cameroon |  |  |  |  |  |  |
| Park *et al.* (2013)(7) | Douala | 2011 | RDS | 255 | 23^†^ (IQR 21–27) | 25.5 (19.1–31.9) ^b^ | 2.2 (1.4–3.0) ͙ͩ |
|  | Yaounde |  |  | 207 | 25^†^ (IQR 21–28) | 44.4 (35.7–53.2) ^b^ | 2.2 (1.7–2.8) ͙ͩ |
|  | Central African Republic | | |  |  |  |  |
| Marcel *et al.* (2019)(8) | Bangui | 2010 | RDS | 99 | 24 | 41.0 (31.4–51.3) ^a^ | 6.2 (3.9–9.7) |
|  | Cote D’Ivoire |  |  |  |  |  |  |
| Hakim *et al.* (2015)(9) | Abidjan | 2011–2012 | RDS | 581 | 23^†^ (IQR 18–51) | 18.0 (13.0–23.1) ^b^ | 3.3 (3.3–3.3) |
| Yaya *et al.* (2021)(6) | Abidjan | 2017-2018 | RDS | 129 | Agegroup 18-24  40.1%,  Agegroup 25-34  51.0%,  Agegroup 35+  8.9% | 32.6 (24.5-40.6) ^a^ | 4.3 (2.0-6.5) |
|  | Eswatini |  |  |  |  |  |  |
| Baral *et al.* (2013)(36) | Manzini | 2011 | RDS | 284 | 23.1 (18–43) | 13.4 (7.9–19.7) ^b^ | 17.6 (15.8–19.4) ͙ͩ |
|  | The Gambia |  |  |  |  |  |  |
| Mason *et al.* (2013)(10) | Banjul | NR | Snowball sampling | 205 | 22 (16–48) | 9.8 (6.2–14.9) ^b^ | 0.6 (0.6–0.6) |
|  | Kenya |  |  |  |  |  |  |
| Muraguri *et al.* (2015)(11) | Nairobi | 2010 | RDS | 290 | > 18 | 12.2 (7.6–17.5) ^b^ | 4.6 (2.4–6.9) ͙ͩ |
| Battacharjee *et al.* (2020)(12) | Kisumu  Mombasa  Kiambu | 2019 | RDS | 1200 | 23 | 16.8 (14.6–18.9) ^a^ | 7.4 (4.9–11.1) |
| Gebrebrhan *et al.* (2020)(13) | Nairobi | 2016 | RDS | 80 | 25^†^ (IQR 23–30) | 42.5 (31.7-53.3) ^a^ | 6.2 (3.5-8.9) |
| Sandfort *et al.* (2019)(15) | Kisumu | 2015-2016 | Sampling frame of MSM organizations | 157 | Agegroup 18-20  8.8%,  Agegroup 21-25  41.8%,  Agegroup 26-44  49.5% | 25.5 (18.7-32.3) | 12.1 (8.5-15.8) |
| Smith *et al.* (2021)(15) | Nairobi | 2017 | RDS | 522 | Agegroup 18-22  39%,  Agegroup 23-29  39%,  Agegroup 30+  23% | 25.0 (21.3-28.7) ^a^ | 5.8 (3.2-8.4) |
|  | Lesotho |  |  |  |  |  |  |
| Stahlman *et al.* (2016)(16) | Maseru | 2014 | RDS | 315 | 22^†^ (IQR 20–26) | 18.0 (12.8–23.2) ^b^ | 29.4 (26.1–32.8) ͙ͩ |
|  | Malawi |  |  |  |  |  |  |
| Wirtz *et al.* (2017)(17) | Blantyre | 2011–2014 | RDS | 338 | 24^†^ | 12.5 (8.5–18.7) ^b^ | 26.5 (22.4–30.6) ͙ͩ |
|  | Chikwawa |  |  | 350 |  | 20.5 (15.7–26.5) ^b^ | 8.6 (8.5–8.7) ͙ͩ |
|  | Mangochi |  |  | 351 |  | 22.4 (17.3–28.5) ^b^ | 15.2 (8.3–22.1) ͙ͩ |
|  | Mulanje |  |  | 349 |  | 24.5 (19.5–30.3) ^b^ | 17.3 (14.8–19.8) ͙ͩ |
|  | Lilongwe |  |  | 362 |  | 17.7 (10.6–28.1) ^b^ | 4.3 (2.7–5.9) ͙ͩ |
|  | Mzuzu |  |  | 353 |  | 4.1 (2.2–7.6) ^b^ | 4.2 (2.2–6.1) ͙ͩ |
|  | Nkhata Bay |  |  | 350 |  | 20.6 (16.3–25.6) ^b^ | 18.9 (12.5–25.3) ͙ͩ |
| Sandfort *et al.* (2019)(15) | Blantyre | 2015-2016 | Sampling frame of MSM organizations | 99 | Agegroup 18-20  5.5%,  Agegroup 21-25  31.2%,  Agegroup 26-44  63.3% | 30.3 (21.2-39.4) | 24.7 (17.4-31.9) ͙ͩ |
|  | Mali |  |  |  |  |  |  |
| Lahuerta *et al.* (2017)(19) | Bamako | 2014–2015 | RDS | 550 | > 18 | 13.7 (9.2–18.1) ^b^ | 1.7 (1.1–2.3) ͙ͩ |
| Kayolta *et al.* (2021)(19) | Bamako | 2019 | RDS | 50 | 24.2 (18-35) | 32.0 (19.1-44.9) ^a^ | 1.5 (0.2-2.9) |
| Yaya *et al.* (2021)(6) | Bamako | 2017-2018 | RDS | 210 | Agegroup 18-24  40.1%,  Agegroup 25-34  51.0%,  Agegroup 35+  8.9% | 32.9 (26.5-39.2) ^a^ | 1.4 (0.1-2.7) |
|  | Mozambique |  |  |  |  |  |  |
| Nalá *et al.* (2015)(20) | Maputo | 2011 | RDS | 496 | 22^†^ | 8.2 (4.7–12.6) ^b^ | 12.8 (11.1–14.5) ͙ͩ |
|  | Beira |  |  | 584 | 21^†^ | 9.1 (5.8–12.6) ^b^ | 11.1 (9.1–13.1) ͙ͩ |
|  | Nampula/  Nacala |  |  | 353 | 21^†^ | 3.7 (1.1–7.1) ^b^ | Nampula: 5.9 (5.9–5.9) ͙ͩ |
|  | Nigeria |  |  |  |  |  |  |
| Keshinro *et al.* (2016)(21) | Abuja | 2013–2016 | RDS | 546 | 24^†^ (IQR  21–27) | 43.5 (37.3–49.6) ^b^ | 2.9 (2.9–2.9) |
|  | Lagos |  |  | 316 |  | 65.6 (54.7–76.5) ^b^ | 1.8 (1.8–1.8) |
| Bamgboye *et al.* (2017)(23) | Eight major cities | 2014 | RDS | 1960 | >15 | 22.2 (20.3–24.1) ^a^ | Kano: 0.6 (0.6–0.6)  Lagos: 1.8 (1.8–1.8)  Cross River: 2.9 (2.9–2.9)  Enugu: 1.5 (1.5–1.5)  Kaduna: 1.8 (1.7–1.8)  Rivers: 4.3 (4.2–4.3)  Oyo: 1.2 (1.2–1.2)  Federal Capital Territory: 2.8 (2.8–2.8) |
| Vu *et al.* (2013)(23) | Abuja | 2010 | RDS | 174 | 25^†^ (18–52) | 34.9 (25.5–45.9) ^c^ | 3.2 (3.2–3.2) |
|  | Ibadan |  |  | 193 | 23^†^ (18–43) | 11.3 (5.1–16.8) ^c^ | 1.4 (1.4–1.4) |
|  | Lagos |  |  | 297 | 21 (18–45) | 15.2 (9.7–21.2) ^c^ | 1.6 (1.6–1.6) |
| Ramadhani *et al.* (2020)(24) | Abuja  Lagos | 2013-2018 | Sampling frame of MSM organization | 1379 | Agegroup 16-19  15.8%,  Agegroup 20-24  42.6%,  Agegroup 25+  41.5% | 47.6 (40.5-54.7) | 4.3 (2.1-6.6) |
|  | Rwanda |  |  |  |  |  |  |
| Ntale *et al.* (2018)(26) | Kigali | 2015 | Snowball sampling | 227 | 23^†^ (IQR 21–26) | 4.4 (2.3–8.2) ^a^ | 5.8 (5.3–6.4) ͙ͩ |
|  | Rubavu |  |  | 64 |  | 7.8 (2.9–18.0) ^a^ | 1.8 (1.4–2.2) ͙ͩ |
|  | Ruhanga |  |  | 62 |  | 4.8 (1.2–14.3) ^a^ | 3.2 (3.2–3.2) ͙ͩ |
|  | Musanze |  |  | 44 |  | 4.6 (0.8–16.8) ^a^ | 1.3 (0.9–1.8) ͙ͩ |
|  | Huye |  |  | 107 |  | 3.7 (5.6–18.2) ^a^ | 3.2 (2.6–3.8) ͙ͩ |
| Murunzi *et al.* (2020)(27) | Kigali | 2016–2017 | Sampling frame of MSM organization | 345 | >18 | 19.4 (15.4–24.1) | 5.8 (5.3–6.4) ͙ͩ |
| Twahirwa Rwema *et al.* (2020)(28) | Kigali | 2018 | RDS | 630 | Agegroup 18-24  46.6%,  Agegroup 25-34  38.4%,  Agegroup 35+  15.1% | 10.2 (7.8-12.6) ^b^ | 5.4 (2.9-7.9) |
|  | Senegal |  |  |  |  |  |  |
| Dramé *et al.* (2013)(29) | Dakar | 2011–2012 | Sampling frame of MSM organizations | 114 | 28 (18–42) | 36.0 (27.4–45.6) ^a^ | 1.1 (0.3–1.9) ͙ͩ |
| Lyons *et al.* (2017)(29) | Dakar  Mbour  Thies | NR | Combination of RDS and purposive sampling | 724 | > 18 | 23.5 (20.5–26.8) ^b^ | 1.1 (0.3–1.9) ͙ͩ  0.0 (0.0–0.0) ͙ͩ  0.1 (0.1–0.1) ͙ͩ |
|  | South Africa |  |  |  |  |  |  |
| Jobson *et al.* (2018)(30) | Townships Cape Town | NR | Chain referral sampling | 153 | 26^†^ (IQR 11) | 30.7 (23.6–38.7) | 7.4 (7.4–7.5) |
|  | Durban |  |  | 69 |  | 27.5 (17.0–38.1) | 10.9 (10.9–10.9) |
| Fearon *et al.* (2017)(32) | Johannesburg | 2017 | RDS | 233 | 26 | 40.8 (34.5–47.4) ^a^ | 16.3 (12.5–21.0) |
| Lane *et al.* (2014)(32) | Gert Sibande district | 2012–2013 | RDS | 195 | > 18 | 28.3 (21.1–35.3) ^b^ | 16.8 (16.7–16.8) |
|  | Ehlanzeni district |  |  | 259 | > 18 | 13.7 (9.1–19.6) ^b^ | 16.8 (16.8–16.9) |
| Sandfort *et al.* (2015)(33) | Tshwane | 2011–2013 | RDS | 480 | 24.5 (18–44) | 30.1 (26.1–34.5) ^b^ | 9.1 (9.0–9.1) |
| Sullivan *et al.* (2020)(34) | Cape Town  Port Elizabeth | 2015-2016 | Sampling frame from MSM organization | 263 | Agegroup 18-19  21.7% CT, 10.2% PE  Agegroup 20-24  34.8% CT,  46.3% PE  Agegroup 25+  34.5% CT,  34.5% PE | 42.0 (36.0-48.0) | 13.1 (9.3-16.8) |
| Sandfort *et al.* (2019)(15) | Cape Town (CT)  Soweto (S) | 2015-2016 | Sampling frame from MSM organization | 140  194 | Agegroup 18-20  23.6% CT,  12.1% S  Agegroup 21-25  41.0% CT,  39.0% S  Agegroup 26-44  35.4% CT,  48.9% | 44.3 (36.1-52.5)  60.9 (54.0-67.8) | 10.9 (7.4-14.4)  16.9 (12.6-21.0) |
|  | Tanzania |  |  |  |  |  |  |
| Ross *et al.* (2014)(36) | Dar es Salaam | 2012 | RDS | 172 | 23^†^ (IQR 21–28) | 30.2 (23.6–37.7) ^a^ | 4.1 (4.1–4.1) |
|  | Tanga | 2013 |  | 90 | NR | 11.1 (5.7–19.9) ^c^ | 2.3 (2.3–2.3) |
| Khatib *et al.* (2017)(37) | Unganja, Zanzibar | 2011 | RDS | 2011:  339 | > 15 | 2011: 2.6 (1.0–4.7) ^c^ | 0.6 (0.6–0.6) |
| Ishungisa *et al.* (2020)(38) | Dar es Salaam | 2017 | RDS | 777 | Agegroup 15-19  17.3%,  Agegroup 20-24  34.8%,  Agegroup 25-29  19.6%,  Agegroup 30-34  13.5%,  Agegroup 35+  14.8% | 8.3 (6.4-10.2) ^b^ | 4.6 (2.2-6.9) |
| Mmbaga *et al.* (2017)(39) | Dodoma municipality | 2014 | RDS | 387 | 27^†^ (8–60) | 17.4 (12.6–25.4) ^b^ | 2.2 (2.2–2.2) |
| Mmbaga *et al.* (2018)(41) | Dar es Salaam | 2014 | RDS | 646 | 26.5 | 22.3 (18.5–26.2) ^b^ | 3.7 (3.7–3.7) |
|  | Togo |  |  |  |  |  |  |
| Holland *et al.* (2016)(5) | Lomé | 2013 | RDS | 354 | > 18 | 18.5 (14.7–23.0) ^a^ | 2.7 (2.1–3.3) ͙ͩ |
|  | Kara |  |  | 329 |  | 0.6 (0.1–2.4) ^a^ | 5.5 (4.3–6.7) ͙ͩ |
| Teclessou *et al.* (2017)(41) | Lome | 2011–2015 | RDS | 215 | 22^†^ (IQR 21–26) | 22.3 (17.0–28.6) ^a^ | 2.72 (2.12–3.32) ͙ͩ |
|  | Eight cities |  |  | 281 | 23^†^ (IQR 21–28) | 5.7 (3.4–9.3) ^a^ | Cinkasse: 2.0 (1.3–2.7) ͙ͩ  Dapaong: 0.0 (0.0–0.0) ͙ͩ  Kara: 5.5 (4.3–6.7) ͙ͩ  Sokode: 2.5 (1.9–3.1) ͙ͩ  Atakpame: 0.0 (0.0–0.0) ͙ͩ  Tsevie: 2.3 (2.3–2.3) ͙ͩ  Kpalime: 2.9 (1.7–4.1) ͙ͩ  Aneho: 1.5 (0.6–2.4) ͙ͩ |
| Tchankoni *et al.* (2020)(42) | Eight cities | 2017 | RDS | 643 | 23 | 21.6 (18.5–25.0) ^b^ | 2.1 (1.5–2.7) ͙ͩ |
| Ferre *et al.* (2019)(44) | Lome  Kpalime  Atakpama  Tsevie | 2017 | RDS | 207 | 22^†^ (IQR 20–26) | 26.1 (20.1-32.1) ^a^ | 2.4 (0.7-4.1) |
| Yaya *et al.* (2021)(6) | Lome | 2017-2018 | RDS | 148 | Agegroup 18-24  40.1%,  Agegroup 25-34  51.0%,  Agegroup 35+  8.9% | 33.1 (25.5-40.7) ^a^ | 2.6 (0.8-4.3) |
|  | Uganda |  |  |  |  |  |  |
| Hladik *et al.* (2017)(44) | Kampala | 2012–2013 | RDS | 607 | 23^†^ (IQR 21–26) | 12.2 (7.9–15.9) ^b^ | 4.5 (4.5–4.5) |
|  | Zimbabwe |  |  |  |  |  |  |
| Parmley *et al.* (2021)(45) | Harare  Bulawayo | 2019 | RDS and chain referral | 416  760 | Agegroup 18-24  46.9%,  Agegroup 25-34  35.4%,  Agegroup 35-44  12.2%,  Agegroup 45+  5.5% | 17.1 (13.5-20.7) ^b^  23.3 (20.3-26.3) ^b^ | 12.4 (8.7-16.0)  16.0 (11.9-20.1) |

For studies that used respondent driven sampling (RDS) to enrol participants, we preferred to retrieve the RDS adjusted prevalence data. If these were not presented, we retrieved crude prevalence estimates.

^†^ Median

^a^ Crude HIV prevalence

^b^ Respondent driven sampling (RDS) adjusted HIV prevalence

^c^ Respondent driven sampling analysis tool (RDSAT) adjusted HIV prevalence

͙ͩ Based on Demographic Health Surveys (DHS)(52) data (others are based on Dwyer-Lindgren et al.(56)).

**Table S4: Overview of the prevalence of HIV infection in transgender women in the included studies and prevalence of HIV in the general male and female population at the study location.**

| **Study** | **Country and location** | **Year(s) of study** | **Recruitment method** | **No. tested for HIV** | **Mean or median and IQR or range of age of the total population** | **HIV prevalence (%)**  **(95% confidence interval)** | **HIV prevalence general population (%) (95% confidence interval)** |
| --- | --- | --- | --- | --- | --- | --- | --- |
|  | Burkina Faso |  |  |  |  |  |  |
| Poteat *et al.* (2017)(46) | Bobo-Dioulasso and Ouagadougou | 2013 | RDS | 108 | 23.68 (16–56) | 3 (0.7–9.2) ^a^ | Bobo-Dioulasso: 2.7 (2.5–2.9) ͙ͩ  Ouagadougou: 2.4 (2.2–2.6) ͙ͩ |
| Stahlman *et al.* (2016)(47) | Bobo-Dioulasso | 2013 | RDS | 75 | 21^†^ (IQR  20–24) | 5.1 (1.9–12.1) ^a^ | Bobo-Dioulasso: 2.7 (2.5–2.9) ͙ͩ  Ouagadougou: 2.4 (2.2–2.6) ͙ͩ |
|  | Ouagandougou |  |  | 23 |  |  |  |
|  | Cote D’Ivoire |  |  |  |  |  |  |
| Poteat *et al.* (2017)(46) | Abidjan, Bouake, Gagnoa, and Yamoussoukro | 2015–2016 | RDS | 298 | 23.68 (16–56) | 26 (21.2–31.4) ^a^ | Abidjan: 4.52 (4.5–4.5)  Bouake: 3.3 (3.3–3.3)  Gagnoa: 2.5 (2.5–2.5)  Yamoussoukro: 3.4 (3.3–3.7) |
| Stahlman *et al.* (2016)(47) | Abidjan, Bouake, Gagnoa, Yamoussoukro | 2015 | RDS | 306 | 23^†^ (IQR 21–27) | 23.5 (18.9–28.7) ^a^ | Abidjan: 4.63 (4.61–4.64)  Bouake: 3.12 (3.12–3.11)  Gagnoa: 2.63 (2.63–2.64)  Yamoussoukro: 3.44 (3.43–3.45) |
|  | Eswatini |  |  |  |  |  |  |
| Poteat *et al.* (2017)(46) | Mbabane | 2011 | RDS | 120 | 23.68 (16–56) | 14.0 (8.6–21.8) ^a^ | 25.5 (24.7–26.3) ͙ͩ |
|  | The Gambia |  |  |  |  |  |  |
| Poteat *et al.* (2017)(46) | Banjul | 2011 | Snowball sampling | 4 | 23.68 (16–56) | 50.0 (9.2–90.8) ^a^ | 1.8 (1.8–1.8) |
|  | Kenya |  |  |  |  |  |  |
| Sandfort *et al.* (2019)(15) | Kisumu | 2015-2016 | Samling frame of MSM organization | 40 | Agegroup 18-20  2.3%,  Agegroup 21-25  35.9%,  Agegroup 26-44  61.5% | 40.0 (24.8-55.2) | 12.1 (8.5-15.8) |
| Smith *et al.* (2021)(15) | Nairobi | 2017 | RDS | 70 | Agegroup 18-22  32.0%,  Agegroup 23-29  49.0%,  Agegroup 30+  23.0% | 41.0 (29.5-52.5) ^a^ | 5.8 (3.2-8.4) |
|  | Lesotho |  |  |  |  |  |  |
| Poteat *et al*. (2017)(46) | Maputsoe  Maseru | 2014 | RDS | 71 | 23.68 (16–56) | 59 (46.7–70.3) ^a^ | 28.6 (27.2–30.1) ͙ͩ  32.4 (30.6–34.3) ͙ͩ |
|  | Malawi |  |  |  |  |  |  |
| Poteat *et al.* (2017)(46) | Lilongwe | 2011–2012 | RDS | 75 | 23.68 (16–56) | 16.0 (8.9–26.7) ^a^ | 7.6 (6.3–8.9) ͙ͩ |
| Sandfort *et al.* (2019)(15) | Blantyre | 2015-2016 | Sampling frame of MSM organization | 50 | Agegroup 18-20  5.5%,  Agegroup 21-25  31.2%,  Agegroup 26-44  63.3% | 48.0 (34.2-61.8) | 15.9 (11.9-20.0) |
|  | Nigeria |  |  |  |  |  |  |
| Ramadhani *et al.* (2020)(24) | Abuja  Lagos | 2013-2018 | Sampling frame of MSM organization | 190 | Agegroup 16-19  15.8%,  Agegroup 20-24  42.6%,  Agegroup 25+  41.5% | 59.5 (56.9-62.1) | 4.3 (2.1-6.6) |
|  | Rwanda |  |  |  |  |  |  |
| Twahirwa Rwema *et al.* (2020)(28) | Kigali | 2018 | RDS | 106 | Agegroup 18-24  39.6%,  Agegroup 25-34  50.9%,  Agegroup 35+  9.5% | 9.4 (3.8-15.0) ^b^ | 5.4 (2.9-7.9) |
|  | Senegal |  |  |  |  |  |  |
| Poteat *et al.* (2017)(46) | Dakar | 2015 | RDS | 199 | 23.68 (16–56) | 39.0 (32.3–46.2) ^a^ | 0.5 (0.0–2.3) |
|  | South Africa |  |  |  |  |  |  |
| Jobson *et al.* (2018)(30) | Cape Town | NR | Chain referral sampling | 18 | 26 (IQR 15–37) | 57.0 (32.6–78.7) ^a^ | 11.0 (10.9–11.1) |
| Fearon *et al.* (2020)(32) | Johannesburg | 2017 | RDS | 44 | 26 | 45.5 (30.7–61.4) ^a^ | 16.3 (12.5–21.0) |
| Sullivan *et al.* (2020)(34) | Cape Town (CT)  Port Elizabeth (PE) | 2015-2016 | RDS | 22 | Agegroup 18-19  21.7% CT, 10.2% PE  Agegroup 20-24  34.8% CT,  46.3% PE  Agegroup 25+  34.5% CT,  34.5% PE | 59.0 (38.4-79.6) ^a^ | 13.1 (9.3-16.8) |
| Sandfort *et al.* (2019)(15) | Cape Town (CT)  Soweto (S) | 2015-2016 | Sampling frame of MSM organization | 43  61 | Agegroup 18-20  23.7% CT,  15.7% S  Agegroup 21-25  10.2% CT,  45.7% S  Agegroup 26-44  66.1% CT,  38.6% S | 60.5 (45.9-75.1)  82.0 (72.4-91.6) | 10.9 (7.4-14.4)  16.8 (12.6-21.0) |
|  | Togo |  |  |  |  |  |  |
| Poteat *et al.* (2017)(46) | Kara  Lomé | 2013 | RDS | 51 | 23.68 (16–56) | 18.0 (9.1–31.8) ^a^ | 4.4 (3.8–5.1) ͙ͩ  4.8 (3.2–6.4) ͙ͩ |
| Stahlman *et al.* (2016)(47) | Kara  Lomé | 2012–2013 | RDS | 49 | 21^†^ (IQR 20–25) | 18.8 (9.6–33.0) ^a^ | 4.4 (3.8–5.1) ͙ͩ  4.8 (3.2–6.4) ͙ͩ |
|  | Zimbabwe |  |  |  |  |  |  |
| Parmley *et al.* (2021)(45) | Harare  Bulawayo | 2019 | RDS and chain referral | 279  56 | Agegroup 18-20  46.9%,  Agegroup 21-25  35.4%,  Agegroup 26-44  12.2%,  Agegroup 45+  5.5% | 28.0 (22.7-33.3) ^b^  25.0 (13.7-36.3) ^b^ | 12.3 (8.6-15.9)  16.1 (12.0-20.1) |

For studies that used respondent driven sampling (RDS) to enrol participants, we preferred to retrieve the RDS adjusted prevalence data. If these were not presented, we retrieved crude prevalence estimates.

^†^ Median

^a^ Crude HIV prevalence

^b^ Respondent driven sampling (RDS) adjusted HIV prevalence

^c^ Respondent driven sampling analysis tool (RDSAT) adjusted HIV prevalence

͙ͩ Based on Demographic Health Surveys (DHS)(52) data (others are based on Dwyer-Lindgren et al.(56)).

**Table S5: Overview of the prevalence of HIV infection in cisgender male sex workers (MSW) in the included studies and prevalence of HIV in the general male population at the study location.**

| **Study** | **Country and location** | **Year(s) of study** | **Recruitment method** | **No. tested for HIV** | **Mean or median and IQR or range of age of the total population** | **HIV prevalence (%)**  **(95% confidence interval)** | **HIV prevalence general male population (%) (95% confidence interval)** |
| --- | --- | --- | --- | --- | --- | --- | --- |
|  | **Kenya** |  |  |  |  |  |  |
| Muraguri *et al.* (2015)(11) | Nairobi | 2010 | RDS | 273 | > 18 | 26.3 (17.8–35.6) ^b^ | 4.6 (2.4–6.9) ͙ͩ |
| McKinnon *et al.* (2014)(48) | Nairobi | 2009–2012 | Hotspot-based and snowball recruitment | 507 | 27^†^ (IQR 24–31) | 40.0 (35.8–44.3) ^a^ | 4.6 (2.4–6.9) ͙ͩ |
| Smith *et al.* (2015)(49) | Mtwapa | NR | Peer referral | 82 | 26^†^ | 25.6 (16.9–36.7) | 1.8 (0.6–3.0) ͙ͩ |
|  | **Nigeria** |  |  |  |  |  |  |
| Bamgboye *et al.* (2017)(23) | Eight major cities | 2014 | RDS | 1212 | > 15 | 17.0 (15.0-19.3) ^a^ | Kano: 0.6 (0.6–0.6)  Lagos: 1.8 (1.8–1.8)  Cross River: 2.9 (2.9–2.9)  Enugu: 1.5 (1.5–1.5)  Kaduna: 1.8 (1.7–1.8)  Rivers: 4.3 (4.2–4.3)  Oyo: 1.2 (1.2–1.2)  Federal Capital Territory: 2.8 (2.8–2.8) |
| Crowell *et al.* (2016)(50) | Abuja  Lagos | 2013-2016 | RDS | 551 | 22^†^ (IQR 20–25) | 50.6 (46.3–54.8) ^a^ | 2.9 (2.9–2.9)  1.8 (1.8–1.8) |

For studies that used respondent driven sampling (RDS) to enrol participants, we preferred to retrieve the RDS adjusted prevalence data. If these were not presented, we retrieved crude prevalence estimates.

^†^ Median

^a^ Crude HIV prevalence

^b^ Respondent driven sampling (RDS) adjusted HIV prevalence

^c^ Respondent driven sampling analysis tool (RDSAT) adjusted HIV prevalence

͙ͩ Based on Demographic Health Surveys (DHS)(52) data (others are based on Dwyer-Lindgren et al.(56)).

**Panel S2: Association between the HIV prevalence in the general population and in key populations.**

For MSM (men who have sex with men) and transgender women (TGW), the total number of studies identified allowed us to extrapolate country and regional specific RRs derived from our meta-analyses to crudely estimate the country specific prevalence and number of people living with HIV aged 15-49 years for both populations for each country in SSA. We first determined the relationship between the study prevalence and general population HIV prevalence for the two populations by fitting a logistic Deming regression, weighted by study size, through all data points, i.e., log odds(y) = α + βx, with x = HIV prevalence in the general population and y = prevalence in MSM or TGW. Only studies with a sample size >80 were included. The resulting regression functions are presented in figure S1 (MSM) and figure S2 (TGW). For MSM and TGW the β coefficient appeared borderline significant (p<0.1) with, p=0.07 and p=0.05. The resulting the functions were: log odds(y) = -1.96 + 0.021x (for MSM), and log odds(y) = -1.64 + 0.059x (for TGW).

In addition, we tested whether year of data collection, legal or illegal status of same sex relationships, and an indicator for the severity of anti LGBT laws(51) could explain some of the observed heterogeneity in the relationship between key- and general population HIV prevalence by testing them as predictors in a logistic regression model.

To estimate the HIV prevalence for MSM and TGW for each country for which we did not have data or did not have sufficient data (number of people tested <80) we then applied the functions to the urban general population HIV prevalence (we used the urban prevalence because all studies were derived from urban areas and thus compared to local urban DHS prevalence). For MSM we used the general population prevalence amongst males aged 15-49, and for transgender women we used the general population prevalence amongst males and females aged 15-49, derived from UNAIDS 2020(55). The general population prevalence was then multiplied by a urban prevalence factor based on the ratio between the general population prevalence and the urban general population prevalence derived from DHS/AIS surveys(52), and for countries without DHS/AIS surveys an average of 1.44 was used, which is the average from all available SSA countries. For the countries for which we had sufficient data, we used the country estimated prevalence in MSM and TGW as presented in the forestplots (Manuscript figure 3-5).

Next, we estimated total country-specific population sizes by applying estimates of the proportion of MSM (range: 1%-4%) and transgender women (range: 0.5%-1%) within populations(54) to the United Nations population size estimates,(57) and determined country-specific absolute HIV burden among by multiplying prevalence estimates with population size estimates.

**Figure S1: Association between the HIV prevalence in the general population and in MSM.** Each black dot represents a unique data point from peer-reviewed studies with at least 80 participants, red dashed line represents the fitted logistic Deming regression used for extrapolation.


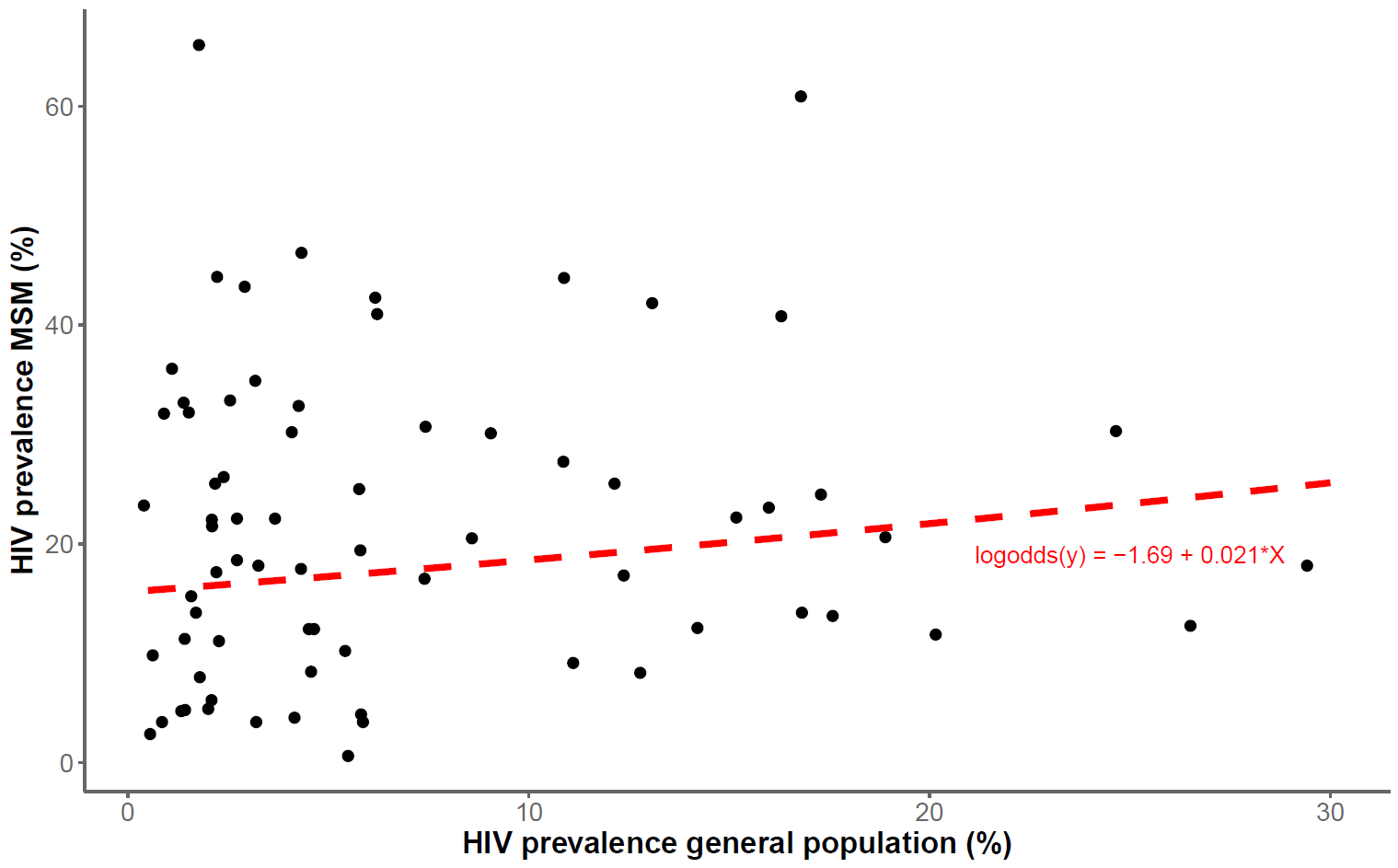


**Table S6: Logistic Deming regression of the relationship between HIV prevalence in the general population and in MSM (used for extrapolation)**

|  | **Exponentiated Coefficient** | **95% CI** | **p-value** |
| --- | --- | --- | --- |
| Intercept | 0.18 | 0.12; 0.27 | <0.0001 |
| HIV prevalence in general pop (continuous) | 1.02 | 0.99; 1.05 | 0.07 |

**Table S7: Univariate logistic regression on relationship between HIV prevalence in MSM and potential confounders**

|  | **Exponentiated Intercept** | **Exponentiated Coefficient** | **95% CI** | **p-value** |
| --- | --- | --- | --- | --- |
| Year of data collection (continuous) | 1.11 e-98 | 1.12 | 1.00; 1.23 | 0.04 |
| Anti LGBT legislation index*  (continuous) | 0.25 | 0.97 | 0.91; 1.05 | 0.53 |
| Legalization of same-sex relations**  (bivariate) | 0.21 | 1.02 | 0.59; 1.76 | 0.92 |

* The anti LGBT legislation index is based on a score from 0-14, with 0 meaning low anti LGBT legislation and 14 meaning high anti-LGBT legislation. Values directly derived from Stannah *et al*. (54).

** Wether same sex relations are legal or illegal. The reference category is “illegal” Values directly derived from Stannah *et al*. (54).

**Figure S2: Univariate Association between the HIV prevalence in the general population and in transgender women (TGW).** Each black dot represents a unique data point from peer-reviewed studies with at least 80 participants, red dashed line represents the fitted logistic Deming regression used for extrapolation.


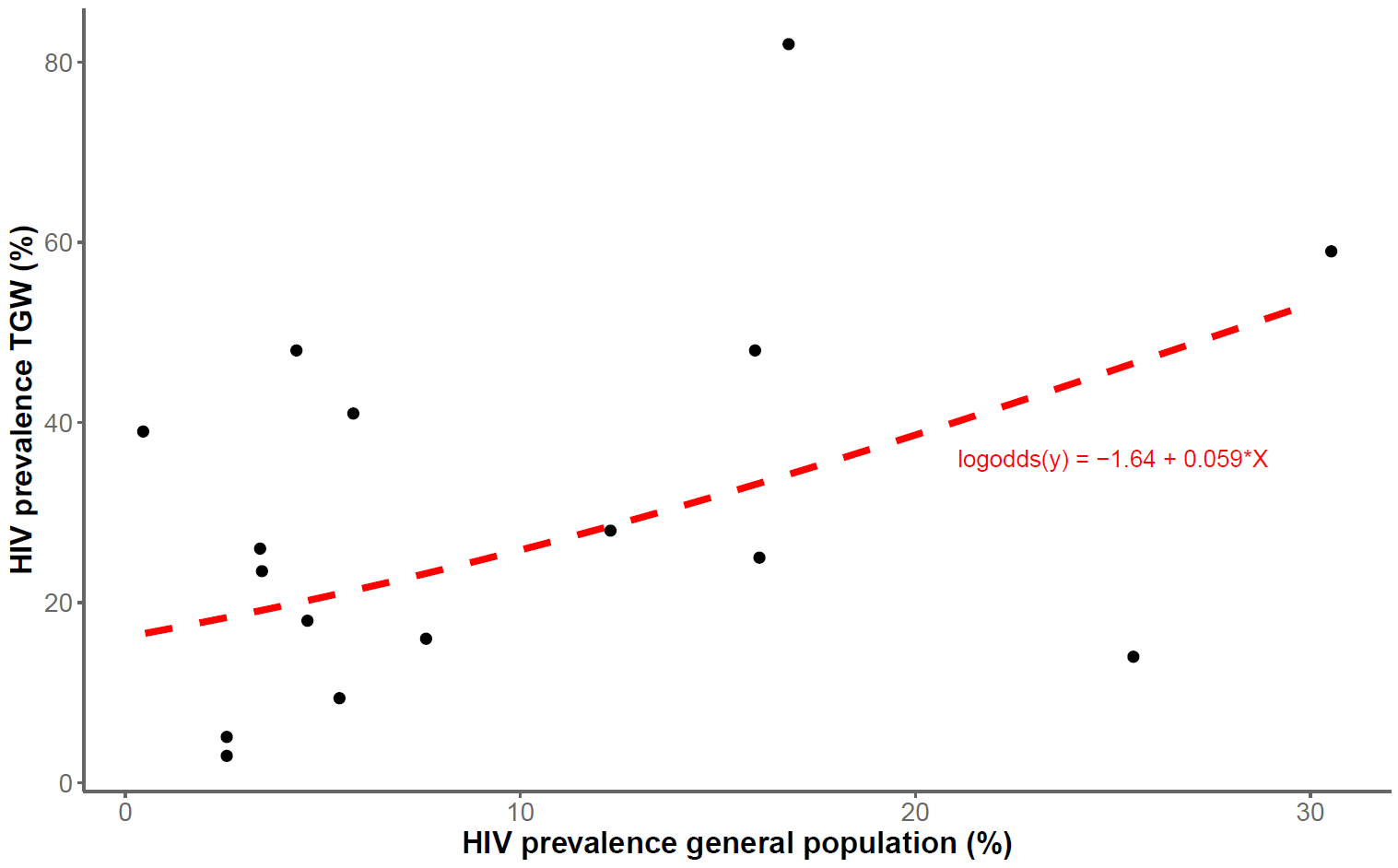


**Table S8: Logistic Deming regression of the relationship between HIV prevalence in the general population and in transgender women (used for extrapolation)**

|  | **Exponentiated Coefficient** | **95% CI** | **p-value** |
| --- | --- | --- | --- |
| Intercept | 0.19 | 0.07; 0.43 | <0.0001 |
| HIV prevalence in general pop (continuous) | 1.06 | 0.99; 1.18 | 0.05 |

**Table S9: Univariate logistic regression on relationship between HIV prevalence in transgender women and potential confounders**

|  | **Exponentiated Intercept** | **Exponentiated Coefficient** | **95% CI** | **p-value** |
| --- | --- | --- | --- | --- |
| Year of data collection (continuous) | 7.65e-137 | 1.17 | 0.88; 1.55 | 0.26 |
| Anti LGBT legislation index*  (continuous) | 0.36 | 0.99 | 0.80; 1.23 | 0.95 |
| Legalization of same-sex relations**  (bivariate) | 0.27 | 2.19 | 0.53; 9.14 | 0.25 |

* The anti LGBT legislation index is based on a score from 0-14, with 0 meaning low anti LGBT legislation and 14 meaning high anti-LGBT legislation. Values directly derived from Stannah *et al*. (54).

** Whether same sex relations are legal or illegal. The reference category is “illegal” Values directly derived from Stannah *et al*. (54).

**Table S10. Review estimations for men who have sex with men (MSM).**

| **Region / Country** |  | **General population males (age 15-49)** | | |  |  | **Men who have sex with men (MSM) Estimations** | |  |
| --- | --- | --- | --- | --- | --- | --- | --- | --- | --- |
|  |  | **HIV prevalence (%)** | **Population size in thousand** | **Source of data for estimation** | | **urban:total HIV prevalence ratio*** | **HIV prevalence MSM (%) (95% confidence interval)** | **Number of MSM living with HIV** |  |
| **Sub-Saharan Africa (SSA)** |  |  |  |  | |  | **20.5** | **600,000 – 2,200,000** |  |
|  |  |  |  |  | |  |  |  |  |
| **Western and central Africa (WCA)** |  | **0.9** |  |  | |  | **22.5** | **300,000 – 1,300,000** |  |
| Angola |  | 1.3 | 7,343 | Study | |  | 3.7 (1.7-5.7) | 3,000 – 11,000 |  |
| Benin |  | 0.6 | 2,888 | Model | | 1.44 ͣ | 15.8 (15.5-16.2) | 5,000 – 18,000 |  |
| Burkina Faso |  | 0.5 | 4,939 | Study | |  | 9.6 (7.6-11.6) | 5,000 – 19,000 |  |
| Cabo Verde |  | 0.5 | 161 | Model | | 1.44 ͣ | 15.8 (15.5-16.1) | <1,000 |  |
| Cameroon |  | 1.9 | 6,489 | Study | |  | 34.0 (29.6-38.3) | 22,000 – 88,000 |  |
| Central African Republic |  | 2.2 | 1,137 | Study | |  | 41.0 (31.3-50.7) | 5,000 – 19,000 |  |
| Chad |  | 0.8 | 3,750 | Model | | 2.69 | 16.2 (15.4-17.2) | 6,000 – 24,000 |  |
| Congo |  | 1.9 | 1,334 | Model | | 1.03 | 16.1 (15.4-17.0) | 2,000 – 9,000 |  |
| Cote d'Ivoire |  | 1.3 | 6,439 | Study | |  | 20.7 (17.7-23.6) | 13,000 – 53,000 |  |
| Democratic Republic of the Congo |  | 0.4 | 19,966 | Model | | 1.33 | 15.7 (15.5-16.0) | 31,000 – 126,000 |  |
| Equatorial Guinea |  | 5.6 | 460 | Model | | 1.44 ͣ | 17.9 (15.0-22.3) | 1,000 – 3,000 |  |
| Gabon |  | 1.6 | 591 | Model | | 1.00 | 16.0 (15.5-16.8) | 1,000 – 4,000 |  |
| Gambia |  | 1.3 | 561 | Study | |  | 9.8 (5.7-13.9) | 1,000 – 2,000 |  |
| Ghana |  | 1.0 | 8,030 | Model | | 1.15 | 15.9 (15.5-16.4) | 13,000 – 51,000 |  |
| Guinea |  | 0.9 | 3,025 | Model | | 1.33 | 15.9 (15.5-16.5) | 5,000 – 19,000 |  |
| Guinea-Bissau |  | 2.2 | 469 | Model | | 1.44 ͣ | 16.5 (15.4-18.0) | 1,000 – 3,000 |  |
| Liberia |  | 0.8 | 1,243 | Model | | 1.37 | 15.9 (15.5-16.4) | 2,000 – 8,000 |  |
| Mali |  | 0.7 | 4,560 | Study | |  | 19.8 (17.1-22.6) | 9,000 – 36,000 |  |
| Mauritania |  | 0.3 | 1,163 | Model | | 1.44 ͣ | 15.7 (15.5-15.9) | 2,000 – 7,000 |  |
| Niger |  | 0.1 | 5,043 | Model | | 2.00 | 15.6 (15.6-15.7) | 8,000 – 32,000 |  |
| Nigeria |  | 1.0 | 48,935 | Study | |  | 33.9 (32.6-35.3) | 166,000 – 664,000 |  |
| Sao Tome and Principe |  | 0.3 | 52 | Model | | 0.53 | 15.6 (15.6-15.7) | <1,000 |  |
| Senegal |  | 0.3 | 3,854 | Study | |  | 25.2 (22.3-28.1) | 10,000 – 39,000 |  |
| Sierra Leone |  | 1.1 | 2,004 | Model | | 1.35 | 16.0 (15.5-16.7) | 3,000 – 13,000 |  |
| Sudan *(and South Sudan)* |  | 0.2 | 13353 | Model | | 1.44 ͣ | 15.7 (15.6-15.8) | 21,000 – 84,000 |  |
| Togo |  | 1.3 | 2,035 | Study | |  | 17.1 (15.6-18.7) | 3,000 – 14,000 |  |
|  |  |  |  |  | |  |  |  |  |
| **East and southern Africa (ESA)** |  | **4.6** |  |  | |  | **18.2** | **200,000 – 900,000** |  |
| Botswana |  | 15.2 | 610 | Study | |  | 13.0 (9.9-16.1) | 1,000 – 3,000 |  |
| Burundi |  | 0.8 | 2,741 | Model | | 2.78 | 16.2 (15.4-17.2) | 4,000 – 18,000 |  |
| Comoros |  | 0.1 | 220 | Model | | 1.44 ͣ | 15.6 (15.6-15.7) | <1,000 |  |
| Djibouti |  | 0.7 | 294 | Model | | 1.44 ͣ | 15.9 (15.5-16.3) | 0 – 2,000 |  |
| Eritrea |  | 0.4 | 846 | Model | | 1.44 ͣ | 15.7 (15.5-16.0) | 1,000 – 5,000 |  |
| Eswatini |  | 18.2 | 296 | Study | |  | 13.4 (9.4-17.4) | 0 – 2,000 |  |
| Ethiopia |  | 0.6 | 28,733 | Model | | 3.22 | 16.1 (15.4-17.0) | 46,000 – 185,000 |  |
| Kenya |  | 2.9 | 13,923 | Study | |  | 19.6 (18.0-21.3) | 27,000 – 109,000 |  |
| Lesotho |  | 16.0 | 588 | Study | |  | 18.0 (13.8-22.2) | 1,000 – 4,000 |  |
| Madagascar |  | 0.3 | 6,822 | Model | | 1.44 ͣ | 15.7 (15.5-15.9) | 11,000 – 43,000 |  |
| Malawi |  | 5.7 | 4,569 | Study | |  | 18.0 (16.5-19.5) | 8,000 – 33,000 |  |
| Mauritius |  | 2.3 | 327 | Model | | 1.44 ͣ | 16.5 (15.4-18.1) | 1,000 – 2,000 |  |
| Mozambique |  | 8.6 | 7,155 | Study | |  | 7.5 (6.1-8.8) | 5,000 – 21,000 |  |
| Namibia |  | 8.3 | 642 | Model | | 0.95 | 16.6 (15.1-22.1) | 1,000 – 4,000 |  |
| Rwanda |  | 1.8 | 3,147 | Study | |  | 10.5 (8.9-12.1) | 3,000 – 13,000 |  |
| Somalia |  | 0.1 | 3,526 | Model | | 1.44 ͣ | 15.6 (15.6-15.7) | 6,000 – 22,000 |  |
| South Africa |  | 13.5 | 16,162 | Study | |  | 36.0 (33.7-38.2) | 58,000 – 233,000 |  |
| Tanzania |  | 3.3 | 14,151 | Study | |  | 14.4 (13.0-15.8) | 20,000 – 82,000 |  |
| Uganda |  | 3.9 | 10,425 | Study | |  | 12.2 (9.6-14.8) | 13,000 – 51,000 |  |
| Zambia |  | 8.0 | 4,394 | Model | | 1.43 | 19.0 (14.8-25.7) | 8,000 – 33,000 |  |
| Zimbabwe |  | 9.1 | 3,391 | Study | |  | 21.1 (18.8-23.4) | 7,000 – 29,000 |  |

ͣ means there was no DHS data available for this country to estimate the urban prevalence factor, therefore we used the mean factor (1.44) based on all available factors from SSA countries.

**Table S11. Review estimations for transgender women (TGW).**

| **Region / Country** |  | **General population (age 15-49)** | | |  | **Transgender women (TGW) Estimations** | |  |
| --- | --- | --- | --- | --- | --- | --- | --- | --- |
|  |  | **HIV prevalence (%)** *(males and females)* | **Population size in thousand** *(females)* | **Source of data for estimation** | **Urban prevalence factor for model** | **HIV prevalence TGW (%) (95% confidence interval)** | **Number of TGW living with HIV** |  |
| **Sub-Saharan Africa (SSA)** |  |  |  |  |  | **28.6** | **400,000 – 800,000** |  |
|  |  |  |  |  |  |  |  |  |
| **Western and central Africa (WCA)** |  | **1.3** |  |  |  | **27.7** | **200,000 – 400,000** |  |
| Angola |  | 1.8 | 7,608 | Model | 1.05 | 17.8 (16-21.0) | 7,000 – 14,000 |  |
| Benin |  | 0.9 | 2,883 | Model | 1.44 | 17.3 (16.1-19.4) | 2,000 – 5,000 |  |
| Burkina Faso |  | 0.7 | 4,891 | Study |  | 4.0 (1.3-6.7) | 1,000 – 2,000 |  |
| Cabo Verde |  | 0.5 | 149 | Model | 1.44 | 16.8 (16.2-18.0) | <1,000 |  |
| Cameroon |  | 3.0 | 6,461 | Model | 1.07 | 19.0 (15.8-25.0) | 6,000 – 12,000 |  |
| Central African Republic |  | 2.9 | 1,141 | Model | 1.44 | 19.9 (15.7-28.1) | 1,000 – 2,000 |  |
| Chad |  | 1.1 | 3,739 | Model | 2.69 | 18.8 (15.9-24.1) | 4,000 – 7,000 |  |
| Congo |  | 3.3 | 1,329 | Model | 1.03 | 19.2 (15.8-25.5) | 1,000 – 3,000 |  |
| Cote d'Ivoire |  | 2.1 | 6,418 | Study |  | 24.7 (21.3-28.2) | 8,000 – 16,000 |  |
| Democratic Republic of the Congo |  | 0.7 | 20,044 | Model | 1.33 | 17.0 (16.1-18.5) | 17,000 – 34,000 |  |
| Equatorial Guinea |  | 7.3 | 312 | Model | 1.44 | 26.5 (14.9-53.0) | <1,000 |  |
| Gabon |  | 3.0 | 552 | Model | 1.00 | 18.8 (15.9-24.3) | <1,000 |  |
| Gambia |  | 1.8 | 582 | Model | 1.00 | 17.7 (16.0-20.8) | <1,000 |  |
| Ghana |  | 1.7 | 7,727 | Model | 1.15 | 17.9 (16.0-21.2) | 7,000 – 14,000 |  |
| Guinea |  | 1.4 | 3,221 | Model | 1.33 | 17.8 (16.0-21.0) | 3,000 – 6,000 |  |
| Guinea-Bissau |  | 3.0 | 492 | Model | 1.44 | 20.0 (15.7-28.5) | <1,000 |  |
| Liberia |  | 1.1 | 1,224 | Model | 1.37 | 17.5 (16.0-20.0) | 1,000 – 2,000 |  |
| Mali |  | 0.9 | 4,541 | Model | 1.73 | 17.5 (16.0-20.1) | 4,000 – 8,000 |  |
| Mauritania |  | 0.3 | 1,138 | Model | 1.44 | 16.6 (16.2-17.3) | 1,000 – 2,000 |  |
| Niger |  | 0.2 | 5,097 | Model | 2.00 | 16.6 (16.2-17.2) | 4,000 – 8,000 |  |
| Nigeria |  | 1.3 | 47,558 | Study |  | 48.0 (40.9-55.1) | 114,000 – 228,000 |  |
| Sao Tome and Principe |  | 0.3 | 52 | Model | 0.53 | 16.4 (16.2-16.6) | <1,000 |  |
| Senegal |  | 0.3 | 4,118 | Study |  | 39.0 (32.2-45.8) | 8,000 – 16,000 |  |
| Sierra Leone |  | 1.5 | 1,967 | Model | 1.35 | 17.9 (16.0-21.4) | 2,000 – 4,000 |  |
| Sudan *(and South Sudan)* |  | 0.2 | 13,401 | Model | 1.44 | 16.5 (16.2-16.9) | 11,000 – 22,000 |  |
| Togo |  | 2.0 | 2,040 | Study |  | 18.4 (10.8-26.0) | 2,000 – 4,000 |  |
|  |  |  |  |  |  |  |  |  |
| **East and southern Africa (ESA)** |  | **6.5** |  |  |  | **29.7** | **200,000 – 400,000** |  |
| Botswana |  | 19.9 | 644 | Model | 1.44 | 51.3 (12.8-95.9) | 2,000 – 3,000 |  |
| Burundi |  | 1 | 2,793 | Model | 2.78 | 18.6 (15.9-23.6) | 3,000 – 5,000 |  |
| Comoros |  | 0.1 | 215 | Model | 1.44 | 16.4 (16.2-16.6) | <1,000 |  |
| Djibouti |  | 0.8 | 257 | Model | 1.44 | 17.2 (16.1-19.0) | <1,000 |  |
| Eritrea |  | 0.5 | 842 | Model | 1.44 | 16.8 (16.2-18.0) | <1,000 |  |
| Eswatini |  | 26.8 | 312 | Study |  | 14.0 (7.8-20.2) | <1,000 |  |
| Ethiopia |  | 0.9 | 28,513 | Model | 3.22 | 18.7 (15.9-24.0) | 27,000 – 53,000 |  |
| Kenya |  | 4.2 | 14,055 | Study |  | 40.6 (31.5-49.8) | 29,000 – 57,000 |  |
| Lesotho |  | 21.1 | 562 | Model | 1.22 | 47.0 (13.1-93.5) | 1,000 – 3,000 |  |
| Madagascar |  | 0.3 | 6,848 | Model | 1.44 | 16.6 (16.2-17.3) | 6,000 – 11,000 |  |
| Malawi |  | 8.1 | 4,707 | Study |  | 28.8 (20.9-36.7) | 7,000 – 14,000 |  |
| Mauritius |  | 1.7 | 322 | Model | 1.44 | 18.3 (15.9-22.6) | <1,000 |  |
| Mozambique |  | 11.5 | 7,559 | Model | 1.27 | 31.5 (14.4-69.2) | 12,000 – 24,000 |  |
| Namibia |  | 11.6 | 670 | Model | 0.95 | 27.1 (14.9-55.1) | 1,000 – 2,000 |  |
| Rwanda |  | 2.5 | 3,300 | Study |  | 9.4 (3.8-15.0) | 2,000 – 3,000 |  |
| Somalia |  | 0.1 | 3,575 | Model | 1.44 | 16.4 (16.2-16.6) | 3,000 – 6,000 |  |
| South Africa |  | 19.1 | 16,091 | Study |  | 63.5 (56.6-70.3) | 51,000 – 102,000 |  |
| Tanzania |  | 4.7 | 14,113 | Model | 1.41 | 22.3 (15.4-37.0) | 16,000 – 31,000 |  |
| Uganda |  | 5.4 | 10,939 | Model | 1.59 | 24.4 (15.2-44.9) | 13,000 – 27,000 |  |
| Zambia |  | 11.1 | 4,507 | Model | 1.43 | 33.1 (14.3-73.5) | 7,000 – 15,000 |  |
| Zimbabwe |  | 11.9 | 3,857 | Study |  | 27.5 (22.7-32.3) | 5,000 – 11,000 |  |

**Table S12. Comparison of UNAIDS estimations versus estimations from this study for men who have sex with men (MSM).** An assessment of whether the point estimate of the HIV prevalence from UNAIDS falls within the HIV prevalence range derived from our study.

| **Men who have sex with men (MSM)** |  | **HIV prevalence UNAIDS (%)** point estimate | **HIV prevalence from this study** category | **Source of data for estimation from this study** | **Comparison** UNAIDS is lower/ equal/  higher compared our study |  |
| --- | --- | --- | --- | --- | --- | --- |
| **Region / Country** |  |  |  |  |  |  |
|  |  |  |  |  |  |  |
| **Western and central Africa (WCA)** |  |  |  |  |  |  |
| Angola |  | 2.0 | <5% | Study | Equal |  |
| Benin |  | 7.0 | 15% - 20% | Model | Lower |  |
| Burkina Faso |  | 1.9 | 5% - 10% | Study | Lower |  |
| Cabo Verde |  |  | 15% - 20% | Model |  |  |
| Cameroon |  | 20.6 | ≥20% | Study | Equal |  |
| Central African Republic |  | 6.5 | ≥20% | Study | Lower |  |
| Chad |  |  | 15% - 20% | Model |  |  |
| Congo |  | 41.2 | 15% - 20% | Model | Higher |  |
| Cote d'Ivoire |  | 7.7 | ≥20% | Study | Lower |  |
| Democratic Republic of the Congo |  | 7.1 | 15% - 20% | Model | Lower |  |
| Equatorial Guinea |  |  | 15% - 20% | Model |  |  |
| Gabon |  |  | 15% - 20% | Model |  |  |
| Gambia |  | 34.4 | 5% - 10% | Study | Higher |  |
| Ghana |  | 18.0 | 15% - 20% | Model | Equal |  |
| Guinea |  | 11.4 | 15% - 20% | Model | Lower |  |
| Guinea-Bissau |  | 3.0 | 15% - 20% | Model | Lower |  |
| Liberia |  | 19.8 | 15% - 20% | Model | Equal |  |
| Mali |  | 12.6 | 15% - 20% | Study | Lower |  |
| Mauritania |  | 23.4 | 15% - 20% | Model | Higher |  |
| Niger |  | 6.4 | 15% - 20% | Model | Lower |  |
| Nigeria |  | 20.9 | ≥20% | Study | Equal |  |
| Sao Tome and Principe |  | 3.2 | 15% - 20% | Model | Lower |  |
| Senegal |  | 27.6 | ≥20% | Study | Equal |  |
| Sierra Leone |  | 14.0 | 15% - 20% | Model | Lower |  |
| Sudan *(and South Sudan)* |  | 0.8 | 15% - 20% | Model | Lower |  |
| Togo |  | 22.0 | 15% - 20% | Study | Higher |  |
|  |  |  |  |  |  |  |
| **East and southern Africa (ESA)** |  |  |  |  |  |  |
| Botswana |  | 14.8 | 10% - 15% | Study | Equal |  |
| Burundi |  | 4.8 | 15% - 20% | Model | Lower |  |
| Comoros |  |  | 15% - 20% | Model |  |  |
| Djibouti |  | 14.2 | 15% - 20% | Model | Lower |  |
| Eritrea |  |  | 15% - 20% | Model |  |  |
| Eswatini |  | 27.2 | 10% - 15% | Study | Higher |  |
| Ethiopia |  |  | 15% - 20% | Model |  |  |
| Kenya |  | 18.2 | 15% - 20% | Study | Equal |  |
| Lesotho |  | 32.9 | 15% - 20% | Study | Higher |  |
| Madagascar |  | 14.9 | 15% - 20% | Model | Lower |  |
| Malawi |  | 12.9 | 15% - 20% | Study | Lower |  |
| Mauritius |  | 17.2 | 15% - 20% | Model | Equal |  |
| Mozambique |  |  | 5% - 10% | Study |  |  |
| Namibia |  | 7.8 | 15% - 20% | Model | Lower |  |
| Rwanda |  | 4.0 | 10% - 15% | Study | Lower |  |
| Somalia |  |  | 15% - 20% | Model |  |  |
| South Africa |  | 18.1 | ≥20% | Study | Lower |  |
| Tanzania |  | 8.4 | 10% - 15% | Study | Lower |  |
| Uganda |  | 13.2 | 10% - 15% | Study | Equal |  |
| Zambia |  |  | 15% - 20% | Model |  |  |
| Zimbabwe |  | 21.1 | ≥20% | Study | Equal |  |

**Table S13. Comparison of UNAIDS estimations versus estimations from this study for transgender women.** An assessment of whether the point estimate of the HIV prevalence from UNAIDS falls within the HIV prevalence range derived from our study.

| **Transgender women** |  | **HIV prevalence UNAIDS (%)** point estimate | **HIV prevalence from this study** category | **Source of data for estimation from this study** | **Comparison** UNAIDS is lower/ equal/  higher compared our study |  |
| --- | --- | --- | --- | --- | --- | --- |
| **Region / Country** |  |  |  |  |  |  |
|  |  |  |  |  |  |  |
| **Western and central Africa (WCA)** |  |  |  |  |  |  |
| Angola |  |  | 15% - 20% | Model |  |  |
| Benin |  | 26.4 | 15% - 20% | Model | Higher |  |
| Burkina Faso |  |  | 0% - 5% | Study |  |  |
| Cabo Verde |  |  | 15% - 20% | Model |  |  |
| Cameroon |  |  | 15% - 20% | Model |  |  |
| Central African Republic |  |  | 15% - 20% | Model |  |  |
| Chad |  |  | 15% - 20% | Model |  |  |
| Congo |  |  | 15% - 20% | Model |  |  |
| Cote d'Ivoire |  | 24.7 | ≥20% | Study | Equal |  |
| Democratic Republic of the Congo |  |  | 15% - 20% | Model |  |  |
| Equatorial Guinea |  |  | ≥20% | Model |  |  |
| Gabon |  |  | 15% - 20% | Model |  |  |
| Gambia |  |  | 15% - 20% | Model |  |  |
| Ghana |  |  | 15% - 20% | Model |  |  |
| Guinea |  |  | 15% - 20% | Model |  |  |
| Guinea-Bissau |  |  | ≥20% | Model |  |  |
| Liberia |  |  | 15% - 20% | Model |  |  |
| Mali |  | 14.6 | 15% - 20% | Model | Lower |  |
| Mauritania |  |  | 15% - 20% | Model |  |  |
| Niger |  |  | 15% - 20% | Model |  |  |
| Nigeria |  | 19.8 | ≥20% | Study | Lower |  |
| Sao Tome and Principe |  |  | 15% - 20% | Model |  |  |
| Senegal |  |  | ≥20% | Study |  |  |
| Sierra Leone |  | 22.4 | 15% - 20% | Model | Higher |  |
| Sudan *(and South Sudan)* |  |  | 15% - 20% | Model |  |  |
| Togo |  |  | 15% - 20% | Study |  |  |
|  |  |  |  |  |  |  |
| **East and southern Africa (ESA)** |  |  |  |  |  |  |
| Botswana |  |  | ≥20% | Model |  |  |
| Burundi |  |  | 15% - 20% | Model |  |  |
| Comoros |  |  | 15% - 20% | Model |  |  |
| Djibouti |  |  | 15% - 20% | Model |  |  |
| Eritrea |  |  | 15% - 20% | Model |  |  |
| Eswatini |  |  | 10% - 15% | Study |  |  |
| Ethiopia |  |  | 15% - 20% | Model |  |  |
| Kenya |  |  | ≥20% | Study |  |  |
| Lesotho |  |  | ≥20% | Model |  |  |
| Madagascar |  |  | 15% - 20% | Model |  |  |
| Malawi |  |  | ≥20% | Study |  |  |
| Mauritius |  | 28.4 | 15% - 20% | Model | Higher |  |
| Mozambique |  |  | ≥20% | Model |  |  |
| Namibia |  |  | ≥20% | Model |  |  |
| Rwanda |  |  | 5% - 10% | Study |  |  |
| Somalia |  |  | 15% - 20% | Model |  |  |
| South Africa |  |  | ≥20% | Study |  |  |
| Tanzania |  |  | ≥20% | Model |  |  |
| Uganda |  |  | ≥20% | Model |  |  |
| Zambia |  |  | ≥20% | Model |  |  |
| Zimbabwe |  | 27.5 | ≥20% | Study | Equal |  |

**Table S14. Comparison of estimations derived from study data to estimations derived regression model for men who have sex with men (MSM).** An assessment of the use of the regression model for studies for which we have study data and whether the estimation from the regression model still falls within the HIV prevalence category range.

| **Men who have sex with men (MSM)** |  | **HIV prevalence regression model (%)** point estimate | **HIV prevalence derived from studies** category | **Comparison** regression model is lower/ equal/  higher compared data derived from studies |  |
| --- | --- | --- | --- | --- | --- |
| **Region / Country** |  |  |  |  |  |
|  |  |  |  |  |  |
| **Western and central Africa (WCA)** |  |  |  |  |  |
| Angola |  | 16.0 | <5% | Higher |  |
| Benin |  |  |  |  |  |
| Burkina Faso |  | 15.9 | 5% - 10% | Higher |  |
| Cabo Verde |  |  |  |  |  |
| Cameroon |  | 16.1 | ≥20% | Lower |  |
| Central African Republic |  | 16.5 | ≥20% | Lower |  |
| Chad |  |  |  |  |  |
| Congo |  |  |  |  |  |
| Cote d'Ivoire |  | 16.0 | ≥20% | Lower |  |
| Democratic Republic of the Congo |  |  |  |  |  |
| Equatorial Guinea |  |  |  |  |  |
| Gabon |  |  |  |  |  |
| Gambia |  | 15.9 | 5% - 10% | Higher |  |
| Ghana |  |  |  |  |  |
| Guinea |  |  |  |  |  |
| Guinea-Bissau |  |  |  |  |  |
| Liberia |  |  |  |  |  |
| Mali |  | 15.9 | 15% - 20% | Equal |  |
| Mauritania |  |  |  |  |  |
| Niger |  |  |  |  |  |
| Nigeria |  | 16.0 | ≥20% | Lower |  |
| Sao Tome and Principe |  |  |  |  |  |
| Senegal |  | 15.6 | ≥20% | Lower |  |
| Sierra Leone |  |  |  |  |  |
| Sudan *(and South Sudan)* |  |  |  |  |  |
| Togo |  | 16.1 | 15% - 20% | Equal |  |
|  |  |  |  |  |  |
| **East and southern Africa (ESA)** |  |  |  |  |  |
| Botswana |  | 22.6 | 10% - 15% | Higher |  |
| Burundi |  |  |  |  |  |
| Comoros |  |  |  |  |  |
| Djibouti |  |  |  |  |  |
| Eritrea |  |  |  |  |  |
| Eswatini |  | 22.7 | 10% - 15% | Higher |  |
| Ethiopia |  |  |  |  |  |
| Kenya |  | 16.5 | 15% - 20% | Equal |  |
| Lesotho |  | 21.8 | 15% - 20% | Higher |  |
| Madagascar |  |  |  |  |  |
| Malawi |  | 18.4 | 15% - 20% | Equal |  |
| Mauritius |  |  |  |  |  |
| Mozambique |  | 18.8 | 5% - 10% | Higher |  |
| Namibia |  |  |  |  |  |
| Rwanda |  | 16.6 | 10% - 15% | Higher |  |
| Somalia |  |  |  |  |  |
| South Africa |  | 19.8 | ≥20% | Lower |  |
| Tanzania |  | 16.9 | 10% - 15% | Higher |  |
| Uganda |  | 17.4 | 10% - 15% | Higher |  |
| Zambia |  |  |  |  |  |
| Zimbabwe |  | 18.4 | ≥20% | Lower |  |

**Table S15. Comparison of estimations derived from study data to estimations derived regression model for transgender women.** An assessment of the use of the regression model for studies for which we have study data and whether the estimation from the regression model still falls within the HIV prevalence category range.

| **Men who have sex with men (MSM)** |  | **HIV prevalence regression model (%)** point estimate | **HIV prevalence derived from studies** category | **Comparison** regression model is lower/ equal/  higher compared data derived from studies |  |
| --- | --- | --- | --- | --- | --- |
| **Region / Country** |  |  |  |  |  |
|  |  |  |  |  |  |
| **Western and central Africa (WCA)** |  |  |  |  |  |
| Angola |  |  |  |  |  |
| Benin |  |  |  |  |  |
| Burkina Faso |  | 17.4 | 0% - 5% | Higher |  |
| Cabo Verde |  |  |  |  |  |
| Cameroon |  |  |  |  |  |
| Central African Republic |  |  |  |  |  |
| Chad |  |  |  |  |  |
| Congo |  |  |  |  |  |
| Cote d'Ivoire |  | 18.3 | ≥20% | Lower |  |
| Democratic Republic of the Congo |  |  |  |  |  |
| Equatorial Guinea |  |  |  |  |  |
| Gabon |  |  |  |  |  |
| Gambia |  |  |  |  |  |
| Ghana |  |  |  |  |  |
| Guinea |  |  |  |  |  |
| Guinea-Bissau |  |  |  |  |  |
| Liberia |  |  |  |  |  |
| Mali |  |  |  |  |  |
| Mauritania |  |  |  |  |  |
| Niger |  |  |  |  |  |
| Nigeria |  | 17.8 | ≥20% | Lower |  |
| Sao Tome and Principe |  |  |  |  |  |
| Senegal |  | 16.4 | ≥20% | Lower |  |
| Sierra Leone |  |  |  |  |  |
| Sudan *(and South Sudan)* |  |  |  |  |  |
| Togo |  | 18.7 | 15% - 20% | Equal |  |
|  |  |  |  |  |  |
| **East and southern Africa (ESA)** |  |  |  |  |  |
| Botswana |  |  |  |  |  |
| Burundi |  |  |  |  |  |
| Comoros |  |  |  |  |  |
| Djibouti |  |  |  |  |  |
| Eritrea |  |  |  |  |  |
| Eswatini |  | 56.9 | 10% - 15% | Higher |  |
| Ethiopia |  |  |  |  |  |
| Kenya |  | 20.5 | ≥20% | Equal |  |
| Lesotho |  |  |  |  |  |
| Madagascar |  |  |  |  |  |
| Malawi |  | 30.0 | ≥20% | Equal |  |
| Mauritius |  |  |  |  |  |
| Mozambique |  |  |  |  |  |
| Namibia |  |  |  |  |  |
| Rwanda |  | 20.8 | 5% - 10% | Higher |  |
| Somalia |  |  |  |  |  |
| South Africa |  | 38.1 | ≥20% | Equal |  |
| Tanzania |  |  |  |  |  |
| Uganda |  |  |  |  |  |
| Zambia |  |  |  |  |  |
| Zimbabwe |  | 28.6 | ≥20% | Equal |  |

**Table S16. Assesment of the impact of the use of geospatially matched Demographic Health Surveys (DHS)(52) data versus Dwyer-Lindgren et al.(56) (DL) data.** An assessment of the use of DL data instead of DHS data on the Prevalence Ratio (PR). Key pop = key population. Gen pop = general population. * Multiple locations and all are included in the analysis, but for overview puposes only the first location is added in this table. See table S3-5 for full study description.

|  |  |  | | |  |  |  |  | |  | |  |
| --- | --- | --- | --- | --- | --- | --- | --- | --- | --- | --- | --- | --- |
| **Study ID** |  | **Population** | **Country** | **Location*** | **HIV prevalence** Study key pop | **HIV prevalence** DHS gen pop | **HIV prevalence** DL gen pop | **PR [CI]** Study **vs** DHS | **PR** Study vs DL | | **Impact**  Using DL data results in a higher/ equal/ lower PR compared to DHS data |  |
|  |  |  |  |  |  |  |  |  |  | |  |  |
| Smith et al. (2015)(49) |  | MSW ESA | Kenya | Mtwapa | 25.6 | 1.8 | 5.8 | 14.3 [2.0-103.5] | 4.4 | | Equal |  |
| Muraguri et al. (2015)(11) |  | MSW ESA | Kenya | Nairobi | 26.3 | 4.6 | 6.9 | 5.7 [2.2-14.3] | 3.8 | | Equal |  |
| McKinnon et al. (2014)(48) |  | MSW ESA | Kenya | Nairobi | 40.0 | 4.6 | 6.2 | 8.6 [3.5-21.4] | 6.5 | | Equal |  |
| Poteat et al. (2017)(46) |  | TGW ESA | Eswatini | Mbabane | 14.0 | 25.5 | 30.1 | 0.5 [0.3-0.9] | 0.5 | | Equal |  |
| Poteat et al. (2017)(46) |  | TGW ESA | Lesotho | Maputsoe* | 59.0 | 30.5 | 27.1 | 1.9 [1.5-2.4] | 2.2 | | Equal |  |
| Poteat et al. (2017)(46) |  | TGW ESA | Malawi | Lilongwe | 16.0 | 7.6 | 10.9 | 2.1 [0.9-4.8] | 1.5 | | Equal |  |
| Poteat et al. (2017)(46) |  | TGW WCA | Burkina Faso | Bobo-Dioulasso* | 3.0 | 2.6 | 1.6 | 1.2 [0.4-3.6] | 1.9 | | Equal |  |
| Stahlman et al. (2016)(47) |  | TGW WCA | Burkina Faso | Bobo-Dioulasso* | 5.1 | 2.6 | 1.6 | 2.0 [0.8-5.0] | 3.2 | | Equal |  |
| Poteat et al. (2017)(46) |  | TGW WCA | Togo | Kara* | 18.0 | 4.6 | 3.1 | 3.9 [2.0-7.5] | 5.9 | | Equal |  |
| Stahlman et al. (2016)(47) |  | TGW WCA | Togo | Kara* | 18.8 | 4.6 | 3.1 | 4.1 [2.1-7.8] | 6.1 | | Equal |  |
| Baral et al. (2013)(36) |  | MSM ESA | Eswatini | Manzini | 13.4 | 17.6 | 29.0 | 0.8 [0.5-1.1] | 0.5 | | Equal |  |
| Muraguri et al. (2015)(11) |  | MSM ESA | Kenya | Nairobi | 12.2 | 4.6 | 6.9 | 2.6 [1.0-6.8] | 1.8 | | Equal |  |
| Stahlman et al. (2016)(16) |  | MSM ESA | Lesotho | Maseru | 18.0 | 29.4 | 27.2 | 0.6 [0.4-0.9] | 0.7 | | Equal |  |
| Wirtz et al. (2017)(17) |  | MSM ESA | Malawi | Blantyre | 12.5 | 26.5 | 18.7 | 0.5 [0.3-0.8] | 0.7 | | Equal |  |
| Wirtz et al. (2017)(17) |  | MSM ESA | Malawi | Mangochi | 22.4 | 15.2 | 13.7 | 1.5 [0.7-3.3] | 1.6 | | Equal |  |
| Wirtz et al. (2017)(17) |  | MSM ESA | Malawi | Mulanje | 24.5 | 17.3 | 20.4 | 1.4 [0.7-3.0] | 1.2 | | Equal |  |
| Wirtz et al. (2017)(17) |  | MSM ESA | Malawi | Lilongwe | 17.7 | 4.3 | 10.2 | 4.1 [0.8-20.3] | 1.7 | | Equal |  |
| Wirtz et al. (2017)(17) |  | MSM ESA | Malawi | Mzuzu | 4.1 | 4.2 | 8.6 | 1.0 [0.2-5.4] | 0.5 | | Equal |  |
| Wirtz et al. (2017)(17) |  | MSM ESA | Malawi | Nkhata Bay | 20.6 | 18.9 | 11.2 | 1.1 [0.5-2.3] | 1.8 | | Equal |  |
| Sandfort et al. (2019)(15) |  | MSM ESA | Malawi | Blantyre | 30.3 | 24.7 | 18.7 | 1.2 [0.8-1.9] | 1.6 | | Equal |  |
| Nalá et al. (2015)(20) |  | MSM ESA | Mozambique | Maputo | 8.2 | 12.8 | 18.3 | 0.6 [0.4-1.0] | 0.4 | | Equal |  |
| Nalá et al. (2015)(20) |  | MSM ESA | Mozambique | Beira | 9.1 | 11.1 | 19.4 | 0.8 [0.4-1.6] | 0.5 | | Equal |  |
| Murunzi et al. (2020)(27) |  | MSM ESA | Rwanda | Kigali | 19.4 | 5.8 | 5.4 | 3.3 [2.1-5.2] | 3.6 | | Equal |  |
| Ntale et al. (2018)(26) |  | MSM ESA | Rwanda | Kigali | 4.4 | 5.8 | 5.6 | 0.8 [0.4-1.6] | 0.8 | | Equal |  |
| Ntale et al. (2018)(26) |  | MSM ESA | Rwanda | Rubavu | 7.8 | 1.8 | 2.8 | 4.3 [1.2-15.2] | 2.8 | | Equal |  |
| Ntale et al. (2018)(26) |  | MSM ESA | Rwanda | Musanze | 4.6 | 1.3 | 2.6 | 3.5 [0.6-19.8] | 1.8 | | Equal |  |
| Ntale et al. (2018)(26) |  | MSM ESA | Rwanda | Huye | 3.7 | 3.2 | 1.9 | 1.2 [0.3-4.2] | 1.9 | | Equal |  |
| Kendall et al. (2014)(3) |  | MSM WCA | Angola | Luande Province | 3.7 | 0.9 | 1.8 | 4.3 [1.1-16.6] | 2.1 | | Equal |  |
| Holland et al. (2016)(5) |  | MSM WCA | Burkina Faso | Ougadougou | 4.7 | 1.3 | 1.4 | 3.5 [1.2-10.4] | 3.5 | | Equal |  |
| Holland et al. (2016)(5) |  | MSM WCA | Burkina Faso | Bobo Dioulasso | 4.9 | 2.0 | 1.9 | 2.4 [0.9-6.9] | 2.6 | | Equal |  |
| Park et al. (2013)(7) |  | MSM WCA | Cameroon | Douala | 25.5 | 2.2 | 4.5 | 11.7 [3.6-38.0] | 5.7 | | Equal |  |
| Park et al. (2013)(7) |  | MSM WCA | Cameroon | Yaounde | 44.4 | 2.2 | 6.1 | 20.0 [9.9-40.4] | 7.3 | | Lower |  |
| Lahuerta et al. (2017)(19) |  | MSM WCA | Mali | Bamako | 13.7 | 1.7 | 1.6 | 8.1 [2.4-27.3] | 8.8 | | Equal |  |
| Dramé et al. (2013)(29) |  | MSM WCA | Senegal | Dakar | 36.0 | 1.1 | 0.5 | 32.8 [5.1-211.8] | 72.4 | | Equal |  |
| Lyons et al. (2017)(29) |  | MSM WCA | Senegal | Dakar* | 23.5 | 0.4 | 0.4 | 58.9 [7.4-470.7] | 60.8 | | Equal |  |
| Holland et al. (2016)(5) |  | MSM WCA | Togo | Lome | 18.5 | 2.7 | 3.4 | 6.8 [3.5-13.2] | 5.5 | | Equal |  |
| Holland et al. (2016)(5) |  | MSM WCA | Togo | Kara | 0.6 | 5.5 | 2.8 | 0.1 [0.0-0.6] | 0.2 | | Equal |  |
| Teclessou et al. (2017)(41) |  | MSM WCA | Togo | Lome | 22.3 | 2.7 | 3.4 | 8.2 [4.2-16.1] | 6.6 | | Equal |  |
| Tchankoni et al. (2020)(42) |  | MSM WCA | Togo | Cinkassé* | 21.6 | 2.1 | 2.0 | 10.3 [6.6-16.1] | 10.7 | | Equal |  |
| Teclessou et al. (2017)(41) |  | MSM WCA | Togo | Cinkassé* | 5.7 | 2.1 | 2.6 | 2.7 [1.4-5.2] | 2.2 | | Equal |  |
| **Overall average PR** |  |  |  |  |  |  |  | **6.1** | **6.2** | |  |  |

**References**

1. African Journals Online. African Journals Online 2022. <https://www.ajol.info/index.php/ajol/Gsearch/google> (accessed 17 March 2022).

2. Ilga Europe. Gossary: ILGA Europe; 2015. <https://www.ilga-europe.org/resources/glossary> (accessed 17 March 2022).

3. Munn Z, Moola S, Lisy K, Riitano D, Tufanaru C. Methodological guidance for systematic reviews of observational epidemiological studies reporting prevalence and cumulative incidence data. Int J Evid Based Healthc. 2015;13(3):147-53.

4. Kendall C, Kerr LRFS, Mota RMS, Cavalcante S, Macena RHM, Chen S, et al. Population size, HIV, and behavior among MSM in Luanda, Angola: Challenges and findings in the first ever HIV and syphilis biological and behavioral survey. J Acquired Immune Defic Syndr. 2014;66(5):544-51.

5. Tafuma TA, Merrigan MB, Okui LA, Lebelonyane R, Bolebantswe J, Mine M, et al. HIV/sexually transmitted infection prevalence and sexual behavior of men who have sex with men in 3 districts of botswana: Results from the 2012 biobehavioral survey. Sex Transm Dis. 2014;41(8):480-5.

6. Holland CE, Kouanda S, Lougué M, Pitche VP, Schwartz S, Anato S, et al. Using population-size estimation and cross-sectional survey methods to evaluate HIV service coverage among key populations in Burkina Faso and Togo. Public Health Rep. 2016;131(6):773-82.

7. Yaya I, Boyer V, Ehlan PA, Coulibaly A, Agboyibor MK, Traoré I, et al. Heterogeneity in the prevalence of high-risk human papillomavirus infection in HIV-negative and HIV-positive men who have sex with men in West Africa. Clin Infect Dis. 2021.

8. Park JN, Papworth E, Kassegne S, Moukam L, Billong SC, Macauley I, et al. HIV prevalence and factors associated with HIV infection among men who have sex with men in Cameroon. J Int AIDS Soc. 2013;16 Suppl 3:18752.

9. Marcel MS, de Dieu LJ, Magloire CPS, Grésenguet G, Ralph-Sydney MB, Piette D, et al. Persistent high-risk behavior and escalating HIV, syphilis and hepatitis b incidences among men who have sex with men living in Bangui, central African republic. Pan Afr Med J. 2018;29.

10. Hakim AJ, Aho J, Semde G, Diarrassouba M, Ehoussou K, Vuylsteke B, et al. The epidemiology of HIV and prevention needs of men who have sex with men in Abidjan, Cote d'Ivoire. PLoS ONE. 2015;10(4).

11. Mason K, Ketende S, Peitzmeier S, Ceesay N, Diouf D, Loum J, et al. A cross-sectional analysis of population demographics, HIV knowledge and risk behaviors, and prevalence and associations of HIV among men who have sex with men in the gambia. AIDS Res Hum Retroviruses. 2013;29(12):1547-52.

12. Muraguri N, Tun W, Okal J, Broz D, Fisher Raymond H, Kellogg T, et al. HIV and STI prevalence and risk factors among male sex workers and other men who have sex with men in nairobi, kenya. J Acquired Immune Defic Syndr. 2015;68(1):91-6.

13. Bhattacharjee P, Isac S, Musyoki H, Emmanuel F, Olango K, Kuria S, et al. HIV prevalence, testing and treatment among men who have sex with men through engagement in virtual sexual networks in Kenya: a cross-sectional bio-behavioural study. J Int AIDS Soc. 2020;23(S2).

14. Gebrebrhan H, Kambaran C, Sivro A, Adhiambo W, Siele N, Becker MG, et al. Rectal microbiota diversity in Kenyan MSM is inversely associated with frequency of receptive anal sex, independent of HIV status. AIDS. 2021;35(7):1091-101.

15. Sandfort TGM, Dominguez K, Kayange N, Ogendo A. HIV testing and the HIV care continuum among sub-Saharan African men who have sex with men and transgender women screened for participation in HPTN …: journals.plos.org; 2019.

16. Smith AD, Kimani J, Kabuti R, Weatherburn P, Fearon E, Bourne A. HIV burden and correlates of infection among transfeminine people and cisgender men who have sex with men in Nairobi, Kenya: an observational study. Lancet HIV. 2021;8(5):e274-e83.

17. Stahlman S, Johnston LG, Yah C, Ketende S, Maziya S, Trapence G, et al. Respondent-driven sampling as a recruitment method for men who have sex with men in southern sub-Saharan Africa: a cross-sectional analysis by wave. Sex Transm Infect. 2016;92(4):292-8.

18. Wirtz AL, Trapence G, Kamba D, Gama V, Chalera R, Jumbe V, et al. Geographical disparities in HIV prevalence and care among men who have sex with men in Malawi: results from a multisite cross-sectional survey. Lancet HIV. 2017;4(6):e260-e9.

19. Lahuerta M, Patnaik P, Ballo T, Telly N, Knox J, Traore B, et al. HIV Prevalence and Related Risk Factors in Men Who Have Sex with Men in Bamako, Mali: Findings from a Bio-behavioral Survey Using Respondent-Driven Sampling. 2017.

20. Koyalta D, Mboumba Bouassa RS, Maiga A, Balde A, Bagendabanga JB, Alinity AA, et al. High Prevalence of Anal Oncogenic Human Papillomavirus Infection in Young Men Who Have Sex with Men Living in Bamako, Mali. Infect Agents Cancer. 2021;16(1).

21. Nalá R, Cummings B, Horth R, Inguane C, Benedetti M, Chissano M, et al. Men who have sex with men in Mozambique: identifying a hidden population at high-risk for HIV. AIDS Behav. 2015;19(2):393-404.

22. Keshinro B, Crowell TA, Nowak RG, Adebajo S, Peel S, Gaydos CA, et al. High prevalence of HIV, chlamydia and gonorrhoea among men who have sex with men and transgender women attending trusted community centres in Abuja and Lagos, Nigeria. J Int AIDS Soc. 2016;19(1).

23. Bamgboye EA, Badru T, Bamgboye A. Transactional Sex between Men and Its Implications on HIV and Sexually Transmitted Infections in Nigeria. J Sex Transm Dis. 2017;2017:1810346.

24. Vu L, Adebajo S, Tun W, Sheehy M, Karlyn A, Njab J, et al. High HIV prevalence among men who have sex with men in Nigeria: Implications for combination prevention. J Acquired Immune Defic Syndr. 2013;63(2):221-7.

25. Ramadhani HO, Crowell TA, Nowak RG, Ndembi N, Kayode BO, Kokogho A, et al. Association of age with healthcare needs and engagement among Nigerian men who have sex with men and transgender women: cross-sectional and longitudinal analyses from an observational cohort. J Int AIDS Soc. 2020;23 Suppl 6(Suppl 6):e25599.

26. Ntale RS, Rutayisire G, Mujyarugamba P, Shema E, Greatorex J, Frost SDW, et al. HIV seroprevalence, self-reported STIs and associated risk factors among men who have sex with men: a cross-sectional study in Rwanda, 2015. 2018.

27. Murenzi G, Kim HY, Munyaneza A, Tuyisenge P, Zawadi TM, Buteera AM, et al. Anogenital Human Papillomavirus and HIV Infection in Rwandan Men Who Have Sex With Men. J Acquir Immune Defic Syndr. 2020;84(5):463-9.

28. Rwema JOT, Lyons CE. HIV infection and engagement in HIV care cascade among men who have sex with men and transgender women in Kigali, Rwanda: a cross‐sectional study. … International AIDS …. 2020.

29. Drame FM, Crawford EE, Diouf D, Beyrer C, Baral SD. A pilot cohort study to assess the feasibility of HIV prevention science research among men who have sex with men in Dakar, Senegal. J Int AIDS Soc. 2013;16 Suppl 3:18753.

30. Lyons CE, Ketende S, Diouf D, Drame FM, Liestman B, Coly K, et al. Potential impact of integrated stigma mitigation interventions in improving HIV/AIDS service delivery and uptake for key populations in senegal. J Acquired Immune Defic Syndr. 2017;74:S52-S9.

31. Jobson G, Tucker A, de Swardt G, Rebe K, Struthers H, McIntyre J, et al. Gender identity and HIV risk among men who have sex with men in Cape Town, South Africa. AIDS Care Psychol Socio-Med Asp AIDS HIV. 2018:1-5.

32. Fearon E, Tenza S, Mokoena C, Moodley K, Smith AD, Bourne A, et al. HIV testing, care and viral suppression among men who have sex with men and transgender individuals in Johannesburg, South Africa. PLoS ONE. 2020;15(6).

33. Lane T, Osmand T, Marr A, Shade SB, Dunkle K, Sandfort T, et al. The mpumalanga men's study (MPMS): Results of a baseline biological and behavioral hiv surveillance survey in two msm communities in south africa. PLoS ONE. 2014;9(11).

34. Sandfort TG, Lane T, Dolezal C, Reddy V. Gender Expression and Risk of HIV Infection Among Black South African Men Who Have Sex with Men. AIDS Behav. 2015;19(12):2270-9.

35. Sullivan PS, Phaswana-Mafuya N, Baral SD, Valencia R, Zahn R, Dominguez K, et al. HIV prevalence and incidence in a cohort of South African men and transgender women who have sex with men: the Sibanye Methods for Prevention Packages Programme (MP3) project. J Int AIDS Soc. 2020;23(S6).

36. Baral SD, Ketende S, Mnisi Z, Mabuza X, Grosso A, Sithole B, et al. A cross-sectional assessment of the burden of HIV and associated individual- and structural-level characteristics among men who have sex with men in Swaziland. J Int AIDS Soc. 2013;16 Suppl 3:18768.

37. Ross MW, Nyoni J, Ahaneku HO, Mbwambo J, McClelland RS, McCurdy SA. High HIV seroprevalence, rectal STIs and riskxy sexual behaviour in men who have sex with men in Dar es Salaam and Tanga, Tanzania. BMJ Open. 2014;4(8).

38. Khatib A, Haji S, Khamis M, Said C, Khalid F, Dahoma M, et al. Reproducibility of Respondent-Driven Sampling (RDS) in Repeat Surveys of Men Who have Sex with Men, Unguja, Zanzibar. AIDS Behav. 2017;21(7):2180-7.

39. Alexander Ishungisa M, Moen K, Leyna G, Makyao N, Ramadhan A, Lange T, et al. HIV prevalence among men who have sex with men following the implementation of the HIV preventive guideline in Tanzania: Respondent-driven sampling survey. BMJ Open. 2020;10(10).

40. Mmbaga EJ, Moen K, Makyao N, Mpembeni R, Leshabari MT. HIV and STI s among men who have sex with men in Dodoma municipality, Tanzania: A cross-sectional study. Sex Transm Infect. 2017;93(5):314-9.

41. Mmbaga EJ, Moen K, Leyna GH, Mpembeni R, Leshabari MT. HIV Prevalence and Associated Risk Factors Among Men Who Have Sex With Men in Dar es Salaam, Tanzania. J Acquir Immune Defic Syndr. 2018;77(3):243-9.

42. Teclessou JN, Akakpo SA, Ekouevi KD, Koumagnanou G, Singo-Tokofai A, Pitche PV. Evolution of HIV prevalence and behavioral factors among MSM in Togo between 2011 and 2015. Pan Afr Med J. 2017;28.

43. Tchankoni MK, Gbeasor-Komlanvi FA, Bitty-Anderson AM, Sewu EK, Zida-Compaore WIC, Alioum A, et al. Prevalence and factors associated with psychological distress among key populations in Togo, 2017. PLoS ONE. 2020;15(4).

44. Ferré VM, Gbeasor-Komlanvi FA. … papillomavirus, human immunodeficiency virus, and other sexually transmitted infections among men who have sex with men in Togo: a national cross-sectional …. Clinical Infectious …. 2019.

45. Hladik W, Sande E, Berry M, Ganafa S, Kiyingi H, Kusiima J, et al. Men Who Have Sex with Men in Kampala, Uganda: Results from a Bio-Behavioral Respondent Driven Sampling Survey. AIDS Behav. 2017;21(5):1478-90.

46. Parmley LE, Chingombe I, Wu Y, Mapingure M, Mugurungi O, Samba C, et al. High burden of active syphilis and HIV/syphilis co-infection among men who have sex with men, transwomen, and genderqueer individuals in Zimbabwe. Sex Transm Dis. 2021.

47. Poteat T, Ackerman B, Diouf D, Ceesay N, Mothopeng T, Odette KZ, et al. HIV prevalence and behavioral and psychosocial factors among transgender women and cisgender men who have sex with men in 8 African countries: A cross-sectional analysis. PLoS Med. 2017;14(11).

48. Stahlman S, Liestman B, Ketende S, Kouanda S, Ky-Zerbo O, Lougue M, et al. Characterizing the HIV risks and potential pathways to HIV infection among transgender women in Cote d'Ivoire, Togo and Burkina Faso. J Int AIDS Soc. 2016;19.

49. McKinnon LR, Gakii G, Juno JA, Izulla P, Munyao J, Ireri N, et al. High HIV risk in a cohort of male sex workers from Nairobi, Kenya. Sex Transm Infect. 2014;90(3):237-42.

50. Smith AD, Muhaari AD, Agwanda C, Kowuor D, Van Der Elst E, Davies A, et al. Heterosexual behaviours among men who sell sex to men in coastal Kenya. AIDS. 2015;29:S201-S10.

51. Crowell TA, Keshinro B, Baral SD, Schwartz SR, Stahlman S, Nowak RG, et al. Stigma, access to healthcare, and HIV risks among men who sell sex to men in Nigeria. J Int AIDS Soc. 2017;20(1).

52. DHS. The DHS program STATcompiler. <http://www.statcompiler.com> (accessed 17 March 2022).

53. Dwyer-Lindgren L, Cork MA, Sligar A, Steuben KM, Wilson KF, Provost NR, et al. Mapping HIV prevalence in sub-Saharan Africa between 2000 and 2017. Nature. 2019;570(7760):189-93.

54. Stannah J, Dale E, Elmes J, Staunton R, Beyrer C, Mitchell KM, et al. HIV testing and engagement with the HIV treatment cascade among men who have sex with men in Africa: a systematic review and meta-analysis. Lancet HIV. 2019;6(11):e769-e87.

55. UNAIDS. Countries: UNAIDS. <https://www.unaids.org/en/regionscountries/countries>; 2020.

56. Caceres CF, Konda K, Segura ER, Lyerla R. Epidemiology of male same-sex behaviour and associated sexual health indicators in low- and middle-income countries: 2003-2007 estimates. Sex Transm Infect. 2008;84 Suppl 1:i49-i56.

57. United Nations. Population: United Nations department of social affairs. <https://www.un.org/en/development/desa/population/publications/database/index.asp> (accessed 17 March 2022).
